# Positively charged cyclodextrins as effective molecular transporters of active phosphorylated forms of gemcitabine into cancer cells

**DOI:** 10.1038/s41598-017-08727-y

**Published:** 2017-08-21

**Authors:** Violeta Rodriguez-Ruiz, Andrey Maksimenko, Giuseppina Salzano, Maria Lampropoulou, Yannis G. Lazarou, Valentina Agostoni, Patrick Couvreur, Ruxandra Gref, Konstantina Yannakopoulou

**Affiliations:** 1 Institut Galien (UMR CNRS 8612), Université Paris-Sud, Université Paris-Saclay, Châtenay-Malabry, France; 2 UMR CNRS 8200, Gustave Roussy, DNA repair group, F-94051 Villejuif, France; 3 Institut des Sciences Moléculaires d’Orsay (UMR CNRS 8214), Université Paris-Sud, Université Paris-Saclay, Orsay, France; 4 National Center for Scientific Research “Demokritos”, Institute of Nanoscience & Nanotechnology, Ag. Paraskevi, 15310 Athens, Greece; 5 Université de Cergy Pontoise, ERRMECe, Biomaterials for Health group, I MAT, F-95302 Cergy, Pontoise France

## Abstract

Positively charged cyclodextrins (PCCDs) are molecular carriers of particular interest for their ability to readily enter into cancer cells. Of main interest, guanidino- and aminoalkyl- PCCDs can be conveniently synthesized and form stable and strong inclusion complexes with various active molecules bearing phosphate groups. We have addressed here the challenge to deliver into cancer cells phosphorylated gemcitabine drugs well known for their instability and inability to permeate cell membranes. NMR data corroborated by semiempirical theoretical calculations have shown that aminoalkyl-CDs form sufficiently stable complexes with both mono- and tri-phosphate forms of gemcitabine by simple mixing of the compounds in aqueous solution at physiological pH. Confocal microscopy and radioactivity counting experiments revealed that the developed systems enabled phosphorylated gemcitabine to penetrate efficiently into aggressive human breast cancer cells (MCF7), eventually leading to a substantial reduction of IC_50_ values. Moreover, compared to free drugs, phosphorylated metabolites of gemcitabine encapsulated in PCCDs displayed improved *in vitro* activities also on the aggressive human cancer cells CCRF-CEM Ara-C/8 C, a nucleoside transport-deficient T leukemia cell line. The current study offers the proof-of-principle that phosphorylated nucleoside drugs could be efficiently transported by PCCDs into cancer cells.

## Introduction

**Figure 1:**
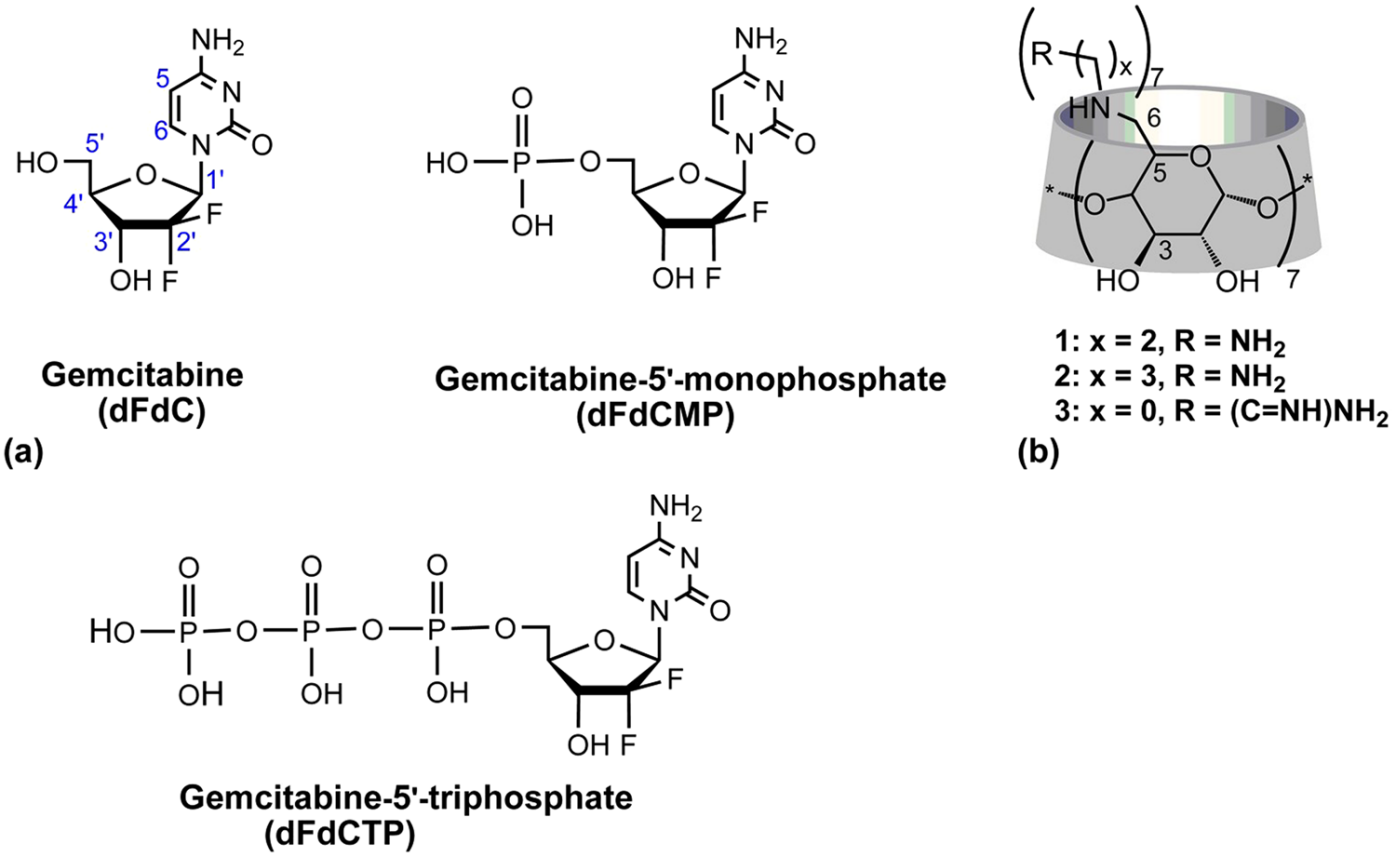
The structures of (**a**) gemcitabine (dFdC), gemcitabine monophosphate (dFdCMP) and gemcitabine triphosphate (dFdCTP) and (**b**) the positively charged βCDs used herein.

Nucleoside analogue prodrugs encompass a range of antiviral and anticancer agents. Among them, the cytidine analogue gemcitabine (2,2′-difluorodeoxycytidine, dFdC) (Fig. 1a) is a first line drug used to treat various solid tumors including non-small-cell lung cancer and pancreatic cancer^1^. Like other nucleoside-derived chemotherapeutics, dFdC relies on nucleoside transporters (NTs) to cross cell membranes^2^. Once internalized, dFdC is converted into gemcitabine monophosphate (dFdCMP) by deoxycytidine kinase (DCK) during a crucial and rate-limiting step^3^. Subsequently, dFdCMP is phosphorylated to the diphosphate (dFdCDP)^4^ and to the active triphosphate (dFdCTP) form^5^ which competes with the natural substrates for incorporation into DNA resulting in inhibition of nucleic acid synthesis and enzymes of nucleotide metabolism^6^. However, development of resistance^7, 8^ and systemic toxicity often occur when intracellular conversion is not efficient. Thus, the direct administration of active triphosphorylated forms of nucleosides, hampered by their poor stability in biological fluids and low cellular uptake, represents a major challenge. Various strategies aimed at increasing the stability and efficiency of active forms of dFdC have been investigated, including their incorporation in colloidal delivery systems as well as their direct conjugation to lipophilic molecules^9–11^. Among the explored strategies, the triphosphated form of dFdC was encapsulated in Lipid/Calcium/Phosphate nanoparticles (LCP)^10^. When intravenously injected, the nanoparticles induced tumor cells apoptosis, reduction of tumor cell proliferation and cell cycle progression, leading to an efficient inhibition of tumor growth. Recently, phosphorylated forms of dFdC were efficiently incorporated into metal-organic frameworks nanoparticles (nanoMOFs) reaching loadings as high as 30 wt%^9^. The nanoMOFs acted as “molecular sponges” soaking the hydrophilic dFdCMP drug molecules from their aqueous solutions. Contrary to free drugs, drug-loaded nanoMOFs showed a significant antiproliferative activity in a pancreatic cancer cell line. However, despite an efficient cell internalization of dFdCMP (of about 6% after only 1 hour incubation), a progressive reduction of the intracellular drug concentration in the following 4 hours of nanoMOFs incubation, suggested possible drug efflux phenomena^9^.

To the best of our knowledge, and as detailed before, only nanoparticulate carriers loaded with phosphorylated dFdC have been studied so far. As an alternative to colloidal nanoparticles, we propose here the use of molecular carriers as delivery systems to transport active phosphorylated dFdC inside cancer cells. In particular, engineered positively charged cyclodextrins (PCCDs) (Fig. 1b) are appealing systems for the delivery of active phosphorylated drugs. Indeed, the ability of PCCDs bearing guanidino and aminoalkylamino groups (*e*.*g*. hosts **1**, **2**, **3**, Fig. 1b) to readily enter cancer cells has been previously demonstrated^12^, highlighting the key role of grafting numerous positive charges on CDs in enhancing cell membrane crossing^13^.

In a previous study, guanidino-CDs (*e*.*g*. host **3** Fig. 1b) have been shown to strongly bind aromatic phosphorylated guests through both cavity inclusion and ionic interactions^14^. Similar binding has been observed for nucleotides^15^ where the deoxyribose moiety was shown to enter the cavity of per(6-guanidino-6-deoxy)-α, -β (**3**) and -γCD in a process driven by the phosphate-guanidino group interactions. On the other hand, the same hosts were unable to bind nucleosides, confirming the importance of electrostatic stabilization for the molecular encapsulation. The capability of amino- or guanidino- substituted PCCDs to host nucleotides has been reported in several similar cases, as recently reviewed^16^. In addition, a p*K*a of 3.6 for the protonated amino group of dFdC has been reported, meaning that at physiological pH dFdC is mostly unprotonated^17^.

In light of these considerations, cell-penetrating PCCD derivatives including per-[6-(2-aminoalkylamino)-6-deoxy]-βCD (hosts **1**, **2**) and per-[6-guanidino-6-deoxy]-βCD (host **3**) (Fig. 1b), which are mostly protonated in neutral aqueous solutions (p*K*a values of host **1** are 6.4 and 9.5; p*K*a values of guanidino-terminated derivative are 7.8 and 11.0^12^) were selected in this study.

The ability of PCCDs to form stable and strong inclusion complexes with phosphorylated forms of dFdC (Fig. 1a)^18^ was investigated by NMR and UV-Vis spectroscopies. Theoretical calculations were performed to provide additional structural insights on the intermolecular interaction of dFdCMP and PCCDs. Finally, the enhanced cellular internalization and *in vitro* cytotoxicity of phosphorylated forms of dFdC when complexed to PCCDs is reported in hormone-dependent breast cancer (MCF7), T cell leukaemia (CCRF-CEM), and nucleoside transport-deficient T cell leukaemia (CCRF-CEM Ara-C/8C) cell lines.

## Results and Discussion

### Complexation studies by NMR spectroscopy

The hosting of dFdC, dFdCMP and dFdCTP in the cavity of hosts **1**, **2** and **3** (Fig. 1b) was studied by NMR spectroscopy in deuterated water and in borate buffer to diminish decomposition of dFdCTP^19^ and also nullify pH effects on the ^1^H and ^19^F chemical shifts^20^. 2D ROESY NMR experiments revealed intermolecular through-space dipolar interactions signifying inclusion between either dFdCMP or dFdCTP and the cavity protons of hosts **1** and **2** (Fig. S1) whereas host **3** interacted only with dFdCMP. The clearly observed interactions involved the CD cavity H3 near the wide opening with protons H5, H6 of the cytosine moiety as well as of H1’ of the difluororibose part of dFdCMP or dFdCTP (numbering shown in Fig. 1a). Table 1 summarizes the observed interactions.

**Table 1:** Host-guest interactions observed in 2D ROESY NMR spectra.

Compound	dFdC	dFdCMP	dFdCTP
1	−*	+*	+*
2		+*	+*
3		+*	−

+Inclusion complex; −no complex; *possible external interactions.

Examination of the ^19^F NMR spectra of the above compositions (Fig. S2) showed that there was a change in the chemical shifts despite the constant pH and concentrations used. This suggests the presence of external intermolecular interactions between the components that possibly creates chemical shift anisotropy effects on the fluorine signals^20^. Additionally, significant broadening of the ^19^F signals was observed, especially of those attributed to the axial F atom, suggesting possible alteration in the relaxation mechanisms and/or conformational exchange. Inclusion complex formation could account for such effects because restriction, for example, of the difluororibose moiety in a cavity could affect its conformational freedom i.e. the *syn*-*anti* conformational exchange or even the rate of C3′-endo (N)/C3′-exo (S) puckering of the ribose, as observed previously for deoxyadenosine monophosphate with **3** ^15^. Extensive broadening was also detected with dFdCMP/**3** and dFdCTP/**3** most likely as the result of external guanidino–phosphate interactions.

A possible mode of cavity inclusion would involve opposite pairing of electric dipoles, i.e. insertion of a guest from the wide CD opening in order to enhance attractive Coulombic forces resulting in stronger binding, partial or total inclusion of the difluororibose moiety as well as part of the nucleobase. The strong stabilizing effect of the phosphate groups leading to unexpected inclusion of the ribose unit in the hydrophobic CD cavity has been demonstrated previously when studying the interaction of nucleotides with **3﻿** ^15^ and also with other amino and guanidino CDs^21–23^. The presence of the two fluorine atoms is expected to increase the hydrophobicity of the ribose moiety and enhance complexation, compared to deoxycytidine or cytidine nucleotides with **3** ^15^.

In summary, both dFdCMP and dFdFTP formed inclusion complexes with the amino-terminated hosts **1** and **2**. Host **1** was selected for the studies hereafter, including theoretical calculations.

### Theoretical Calculations on the Intermolecular Interactions of dFdCMP with host **1**

Theoretical calculations were carried out at the semiempirical PM7 level of theory, including the treatment of solvent effects by the COSMO methodology, for dFdCMP, **1** and their complexes. Extensive conformational analyses for all dominant ionization states in aqueous solution for both dFdCMP and **1** were performed. Pertinent details are described in the supporting information section.

**Figure 2:**
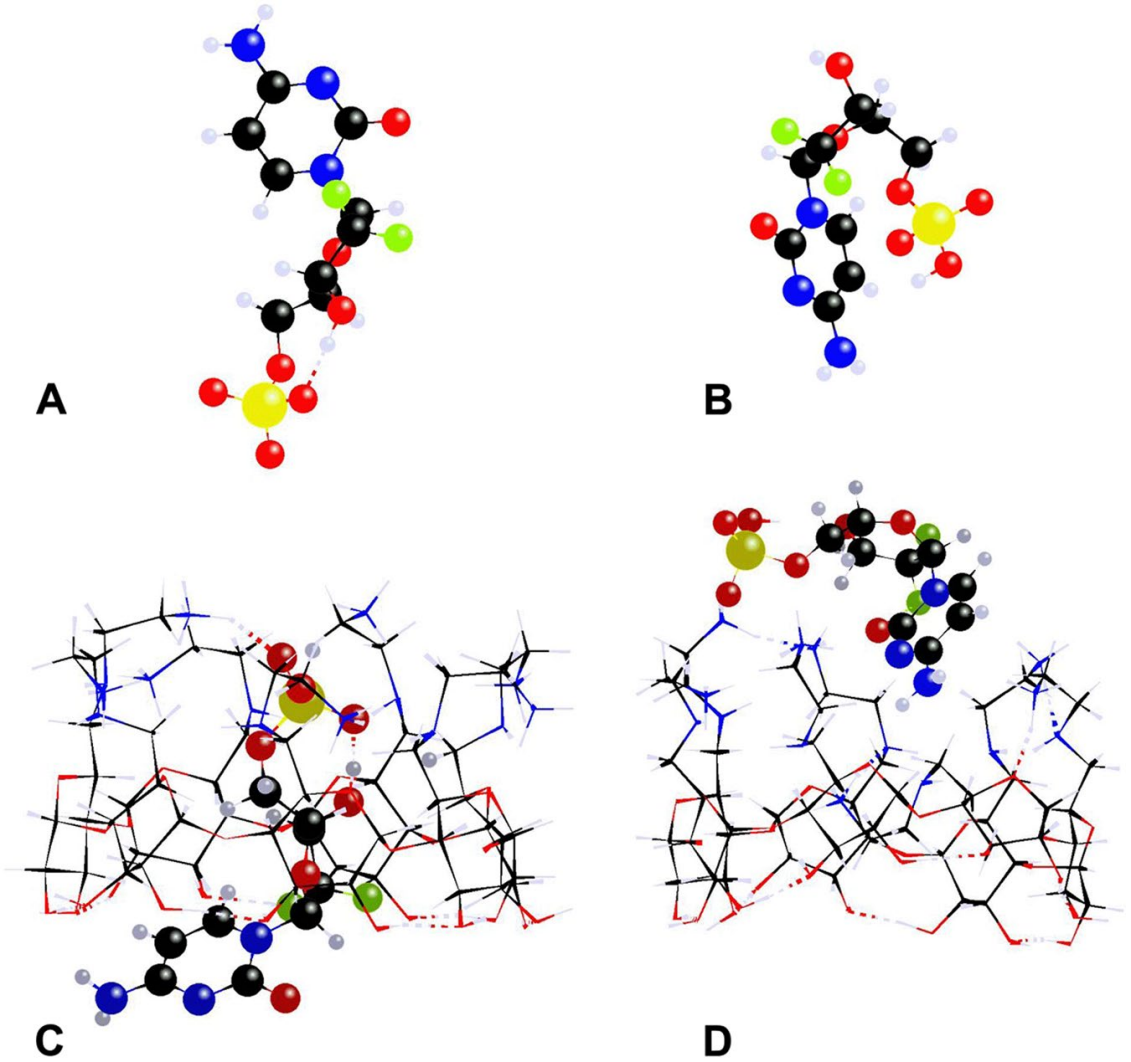
The lowest-energy conformers of dFdCMP^−2^ (**A**, gg) and dFdCMP^−1^ (**B**, tg) and the most stable complexes of **1**H_7_^7+^ via inclusion of dFdCMP^−1^ (**C**) and external association of dFdCMP^−2^ (**D**) at the PM7-COSMO level of theory. Color code: C (black); H (off-white); N (blue); O (red); F (green); P (dark yellow).

For dFdCMP the *syn*- and *anti*- conformations for cytosine and the C3′-endo (N) or the C2′-endo (S) puckering of the ribose ring were considered, as well as all three possible staggered conformations around the C4′-C5′ axis, yielding the corresponding gauche-gauche (gg), gauche-trans (gt) and trans-gauche (tg) conformers, which affected the compactness of the dFdCMP molecule and the location of the phosphate group relative to the ribose unit. In addition, the internal rotation around the C5′-O_P_ axis and the resultant orientation of the phosphate group was also considered. By examining the pKa values for structurally similar species^15, 17, 24^ (see supporting information), the pKa values of dFdCMP were estimated to be 0.7, 6.55 and 3.9 for the phosphate and cytosine moieties, respectively. Therefore, at a pH of 7.3 the guest molecule exists primarily as the dianion dFdCMP^−2^ (85%) with a smaller percentage of the monoanion dFdCMP^−1^ (15%). In regard to their overall structure, dFdCMP^−2^ tends to adopt less compact configurations (Figs 2A and S3A,B) than dFdCMP^−1^ (Figs 2B and S3C,D), attributed to the more efficient solvation of the doubly ionized phosphate group by water molecules. It was found that most low-lying conformers of dFdCMP^−1^ possess the more compact gt and tg conformations, where the hydrogen atom of the phosphate group makes intermolecular H…N and H…O hydrogen bonds.

Host **1** was calculated to exist primarily as the cation **1**H_7_^7+^ at pH of 7.3, protonated on the primary amino groups (see details in supporting information section) in accordance with a previous experimental determination of its acidity constants^12^. Protonation of the primary amino groups was more stable than protonation of the secondary amino groups by 76 kJ mol^−1^.

The most stable species possesses a symmetric all-gg conformation (Fig. S4A) and lies only 0.4 kJ mol^−1^ lower in energy than the most stable mixed gt/tg conformer which contains a -CH_2_NHCH_2_CH_2_NH_3_^+^ chain located nearly inside the macrocycle (Fig. S4B). Mutual electrostatic repulsions are effectively moderated by the high dielectric constant of water and furthermore compensated by the formation of intramolecular -N-H…N- and -N-H…O- hydrogen bonds.

Complexes of dFdCMP with **1**H_7_^7+^ may be formed either by external association on the positively charged primary side or by inclusion via the secondary side of the CD cavity in a mode that maximizes electrostatic attractions. Association complexes are formed with the mixed gt/tg conformers of **1**H_7_^7+^ owing to the higher density of -NH_3_^+^ groups. The stability of inclusion complexes was calculated to be greater for mixed gg/gt than all-gg conformers of **1**H_7_^7+^, due to the favorable proximity of phosphate group to -NH_3_^+^ groups on side chains possessing the gt conformation. However, the reaction enthalpies for the two types of complexes are similar (within 10 kJ mol^−1^), suggesting that both may occur in solution, with calculated exothermicities reaching −160 kJ mol^−1^ for dFdCMP^−1^ and −175 kJ mol^−1^ for dFdCMP^−2^. The corresponding geometries of the most stable complexes are shown in Fig. 2C and D. Additional low energy structures of complexes are shown in Fig. S5, including a low energy inclusion complex of dFdCMP^−2^. All inclusion complexes (Figs 2 and S5) are formed with the difluororibose moiety in the cavity establishing H-bonds with the amino hydrogen atoms further stabilized by electrostatic attractions. The cytosine part lies adjacent to the wider side of the cavity.

**Figure 3:**
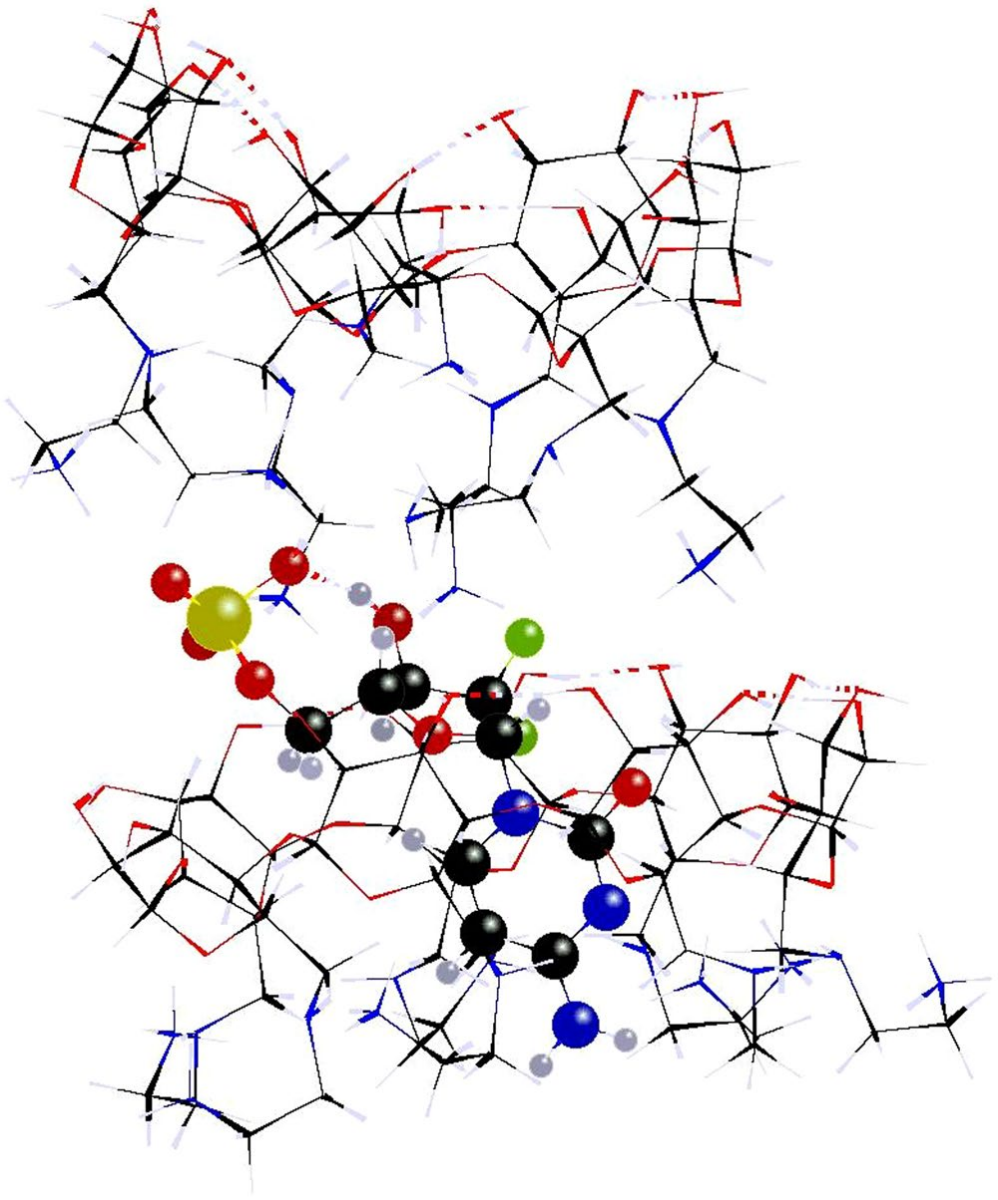
Most stable 1:2 complex of dFdCMP^−2^ with two molecules of **1**H_7_^7+^. Color code: C (black); H (off-white); N (blue); O (red); F (green); P (yellow).

The formation of 1:2 complexes of dFdCMP^−2^ with 1H_7_^7+^ by electrostatic association of the phosphate group on the primary side of a mixed gt/tg **1**H_7_^7+^ and a simultaneous inclusion of the cytosine moiety in the cavity of another all-gg **1**H_7_^7+^ with an even higher exothermicity of −237 kJ mol^−1^ may also occur, as experimentally found in complexes of dCMP/**3** in solution^15^. The geometry of the most stable 1:2 complex is shown in Fig. 3.

The minimum distances (<3.5 Å) between hydrogen atoms H1′, H5 and H6 of dFdCMP and hydrogen atoms CDH3 and CDH5 in the cavity of **1**H_7_^7+^ were computed for the most stable 1:1 and 1:2 complexes of dFdCMP with **1**H_7_^7+^ (Table S1). The resulting large inter-proton distances suggest that the external association complexes should not exhibit NMR-observable through- space dipolar interactions with the interior of **1**H_7_^7+^ in solution. However, the inclusion complexes should display through-space interactions because the internal protons of **1**H_7_^7+^ can be closer than 3 Å from dFdCMP protons (Table S1). Indeed, the correlations observed in 2D ROESY NMR experiments may be attributed to the formation of inclusion complexes of dFdCMP^−2^, considering its dominance in a neutral aqueous solution.

### Stability studies of phosphorylated dFdC forms in complex with host **1**

The binding constants for dFdCMP**/1** at ambient temperature and at 37 °C in phosphate buffer saline (PBS) were obtained from UV–Vis titrations of dFdCMP with excess host **1**. Linear fitting based on 1:1 stoichiometry (prevailing, as indicated by the theoretical results) provided indicative binding constants, K = 7.0 × 10^3^ M^–1^ (R^2^ = 0.995, 22 °C) and 1.4 × 10^3^ M^−1^ R^2^ = 0.966, 37 °C) (Figure S6), suggesting significant binding between the components at human body temperature. The phosphate buffer is expected to influence negatively the strength of binding since it would predictably compete with dFdCMP toward binding with host **1**, however, the value of K remained appreciable.

**Figure 4:**
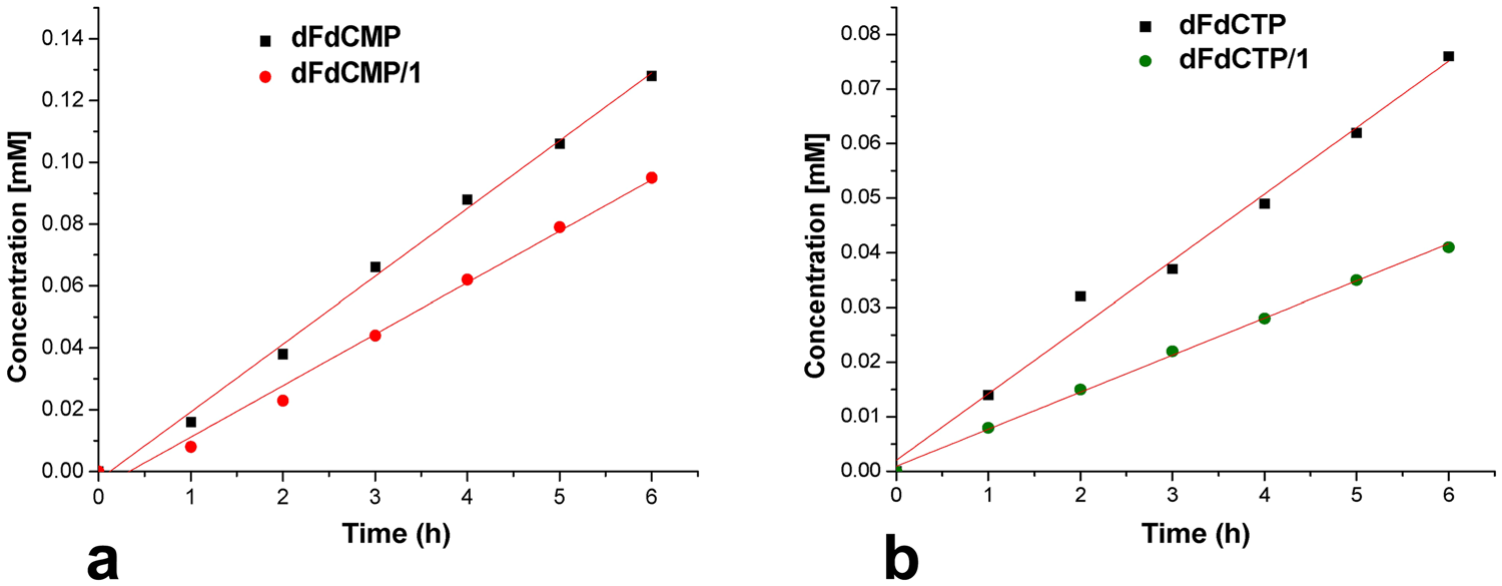
Diffusion experiments. Concentration *vs* time, determined at 37 °C, PBS, pH 7.4 of (**a**) dFdCMP alone (1 mM, black circles, R^2^ = 0.9958) and in the presence of **1** (1:1 molar ratio, red circles, R^2^ = 0.9898) and (**b**) dFdCTP alone (1 mM, black circles, R^2^ = 0.9878) and in the presence of **1** (1:2.5 molar ratio, green circles, R^2^ = 0.9980).

The diffusion of dFdCMP and dFdCTP in the presence of **1** in PBS at 37 °C was evaluated using a modified Franz cell setup. The effect of **1** was assessed by comparing with the blank experiments performed with the guest alone. It was found that **1** reduced the diffusion rate of both dFdCTP and dFdCMP, confirming the considerable host/guest association under the experimental conditions. Specifically, the diffusion rate of dFdCMP was decreased by 24% at 1:1 guest:host ratio (Fig. 4a), while that of dFdCTP was decreased by 44% (Fig. 4b) at a 1:2.6 guest:host ratio. The latter difference may be rationalized by the larger size and the higher number of anionic groups in dFdCTP, also reflecting a stoichiometry different than 1:1.

### MCF7 cell internalization studies by confocal microscopy and flow cytometry

**Figure 5:**
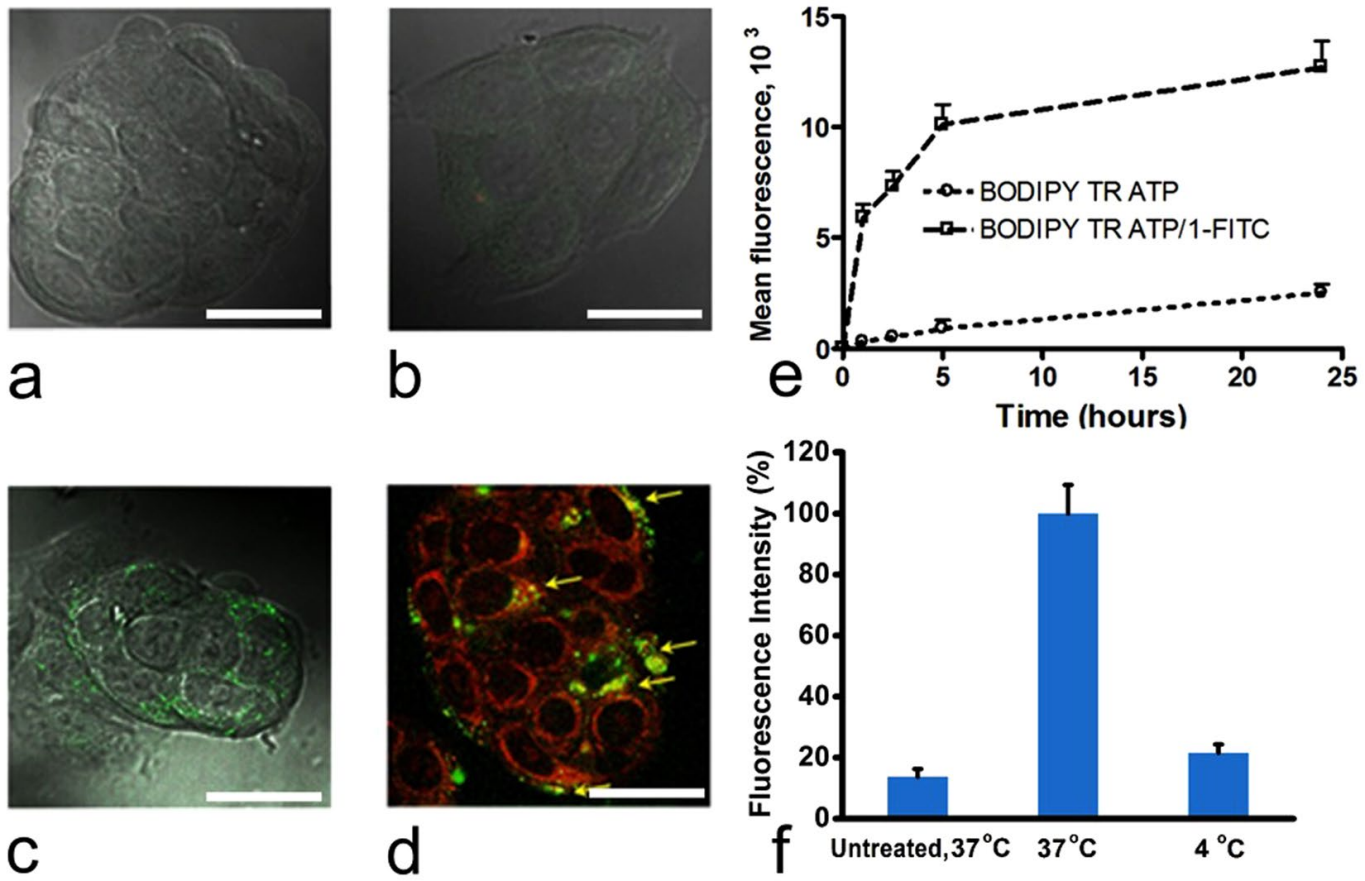
Confocal microscopy images of MCF7 cancer cells incubated 24 h at 37 °C (**a**) without treatment, merged image of contrast phase with green fluorescence (**b**) BODIPY® TR ATP alone, merged image of contrast phase with red fluorescence, **1-FITC** alone, merged image of contrast phase with green fluorescence (**d**) BODIPY® TR ATP**/1-FITC** complex, merged image of red and green fluorescence: arrows indicate yellow spots of clear red and green fluorescence co-localization. Scale bar = 20 microns. Kinetics of cellular uptake of BODIPY® TR ATP encapsulated (or not) in CDs in MCF7 cells (**e**). (**f**) Energy-dependent internalization of BODIPY® TR ATP**/1-FITC** complex in MCF7 cells (mean fluorescence ± SD). Cells were treated with 1 µM of BODIPY® TR ATP/**1-FITC** complex at either 37 °C or 4 °C or untreated (negative control). Results were normalized with respect to the data obtained at 37 °C.

The ability of host **1** to transport hydrophilic phosphorylated molecules inside cancer cells has been studied using the MCF7 breast cancer cell line. Studies were carried out using fluorescein isothiocyanate labeled host, **1-FITC** ^12^ and a model triphosphate compound, BODIPY® TR ATP (commercial labeled nucleotide) for detection of green and red emission, respectively. Cell fluorescence was analysed by a combination of flow cytometry and confocal microscopy, and compared to untreated cells or to cells treated by BODIPY® TR ATP alone. Thus, MCF7 cells were incubated with BODIPY® TR ATP alone or in a 1:1 complex with **1-FITC** (structures shown in Fig. S7) for 24 h and subsequently were examined by confocal microscopy (Fig. 5a–d) after 24 h of cell incubation or by flow cytometry (Fig. 5e) after cell exposure for 1, 2.5, 5 and 24 h.

As expected, no red fluorescence was detected in the cells after incubation with BODIPY® TR ATP (Fig. 5b), confirming the low cellular penetration of this phosphorylated hydrophilic compound. The detected fluorescence was similar to the autofluorescence of untreated cells (Fig. 5a). Contrary to BODIPY® TR ATP, **1-FITC** alone could efficiently penetrate inside the cells and apparently localized inside subcellular compartments (Fig. 5c) as indicated by the spotted green fluorescence observed. Remarkably, when using BODIPY® TR ATP/**1-FITC** complexes under red fluorescence detection (Fig. 5d), extensive emission of BODIPY® TR ATP was observed, mostly spread in the cytosol.

Co-localization of BODIPY® TR ATP and its carrier **1-FITC** was shown by the detection of yellow dotted regions in the overlaid red and green image (Fig. 5d, arrows). This suggests that the complex BODIPY® TR ATP**/1-FITC** was transported intact inside the cells and consequently, during the 24 hours of incubation period, a large part of BODIPY® TR ATP was released into the cytosol, escaping from the cavity of **1-FITC** and the subcellular confinement. These observations were confirmed by flow cytometry experiments which showed a marked enhancement of cell internalization in MCF7 cells (Fig. 5e), with for instance, a 5-fold increase of encapsulated BODIPY® TR ATP in **1**-FITC at 24 h. Furthermore, the cell penetration of BODIPY® TR ATP**/1-FITC** was faster and the intracellular drug concentration remained greater than BODIPY TR ATP alone as early as 1 h post incubation. When the cells were incubated with BODIPY® TR ATP/**1-FITC** (1 μM) for 5 h at 4 °C (instead of 37 °C), the cell fluorescence intensity dramatically decreased (Fig. 5f), suggesting endocytosis as the major pathway of cell internalization of studied complexes.

In a nutshell, the confocal microscopy results indicate the clear and essential role of host **1** to increase the intracellular concentration of the model triphosphorylated hydrophilic BODIPY® TR ATP into cancer cells. These findings suggest that the PCCD host **1** can promote efficient complexation with phosphorylated drugs, enabling the entire complexes to cross the cell membranes and efficiently deliver their cargo inside the cells.

### Internalisation of ^3^H- dFdC/**1** complexes in cancer cells

**Figure 6:**
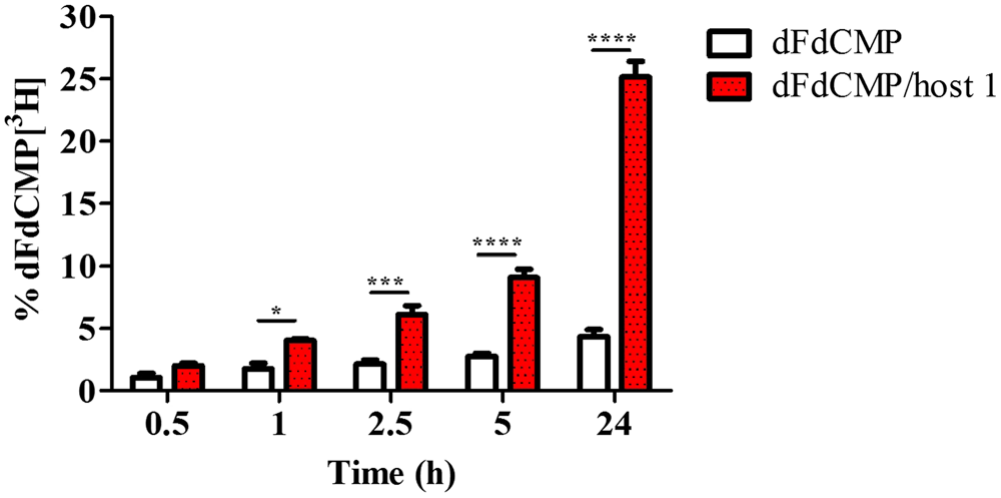
Cellular internalization of radiolabeled dFdCMP in MCF7 cells, free (white) or complexed with host **1** (red). ****p < 0.0001, ***p < 0.005, *p < 0.05, one-way ANOVA with Bonferroni correction.

The rate of dFdCMP internalization into MCF7 cells was comparatively assessed using the free form of tritiated dFdCMP and the same amount of complexed drug with host **1** (Fig. 6). The use of radioactive drugs has two major advantages: (i) active dFdCMP is used and not a chemically modified derivative with possible alteration of physicochemical properties, and (ii) quantitative data can be obtained.

As expected, dFdCMP poorly penetrated inside the cancer cells, as less than 4% of the drug was internalized even after 24 hours of incubation (Fig. 6). In contrast, when dFdCMP was complexed with host **1**, a significant enhancement of the drug internalization was observed starting from the first hour of incubation and reaching a 5-fold increase of dFdCMP internalization after 24 hours of incubation. These findings were in agreement with the confocal microscopy results and confirmed the essential role of host **1** in enabling the cellular delivery of the active phosphorylated forms of dFdC.

It is worth to note that, by using cell penetrating PCCDs to deliver dFdCMP, a progressive increase of the cellular internalization of dFdCMP was achieved following incubation from 1 to 24 hours (Fig. 6).

### Biological studies of dFdC phosphorylated forms complexed to host **1**

Finally, the proof of concept was given by measuring *in vitro* the anticancer efficacy of active phosphorylated dFdCMP and dFdCTP as complexes with PCCD host **1** *versus* the free compounds. Thus, the half maximal inhibitory concentration (IC_50_) of the cell proliferation was measured on three human cancer cell lines with varying genetic status and sensitivity to dFdC: i) hormone-dependent breast cancer (MCF7) ii) T cell leukaemia (CCRF-CEM) and 3) nucleoside transport-deficient T cell leukaemia (CCRF-CEM Ara-C/8 C) cell lines. As a general trend, the phosphorylated forms of dFdC complexed with the PCCD host **1** showed improved anticancer activity on all tested cell lines compared to a control series of the free drugs (Table 2) or host **1** alone (Supporting Information, Fig. S8) at concentrations up to 42 μM. The results for MCF7 cells after 72 h of incubation were consistent using two different assays, MTT and CellTiter-Blue®. No significant difference was observed in the parent leukaemia CCRF-CEM cell line. Interestingly, phosphorylated forms of dFdC complexed with host **1** displayed increased anticancer activity in the nucleoside transport-deficient CCRF-CEM Ara-C/8 C cells as compared to free drugs (Table 2). Equilibrative nucleoside transporter (ENT)-mediated transport of nucleoside analogues is often the rate-limiting step for drug-induced cell cytotoxicity^25^. In these studies, host **1** was indispensable for the cellular internalization of the phosphorylated forms of dFdC, achieving a significant enhancement of the cytotoxic activity of the drugs without toxicity of the transporting host **1**(Fig. S8)

**Table 2:** *In vitro* cytotoxic activities (IC_50_*, µM) on human cancer cell lines of free gemcitabine (dFdC) and its active phosphorylated forms (dFdCMP and dFdCTP) free or encapsulated in host **1**.

Compound	MCF7 MTT	MCF7 CellTiter-Blue®	CCRF-CEM	CCRF-CEM Ara-C/8C
dFdC	21 ± 0.8	16.1 ± 0.1	0.018 ± 0.007	7.4 ± 0.5
dFdCMP	29 ± 0.7	>35	0.033 ± 0.002	11.8 ± 0.7
dFdCTP	>36	>35	0.030 ± 0.003	10.9 ± 0.7
dFdCMP/host **1**	3.5 ± 0.5	2.0 ± 0.1	0.010 ± 0.005	3.3 ± 0.2
dFdCTP/host **1**	3.2 ± 0.6	2.7 ± 0.1	0.027 ± 0.002	1.2 ± 0.1

*The value of IC_50_ was calculated from the dose–response curve presented as dependence of cell viability on the concentration of studied compounds.

## Conclusion

This work generated a new molecular carrier to deliver the challenging phosphorylated forms of gemcitabine into cancer cells. Cell-penetrating PCCDs formed strong and stable inclusion complexes with active dFdCMP and dFdCTP by simple mixing the compounds in aqueous solution. Moreover, these complexes enabled the efficient intracellular capture of the active forms of dFdC in an aggressive cancer cell line, where uptake of the active drugs alone was poor, resulting in significant increase of efficacy. Moreover, our *in vitro* studies showed that the PCCD derivatives allowed increasing the amount of drugs transported inside the cells by endocytosis, an energy-dependent mechanism, and accelerating the cell death by accumulation of drugs in the cells. Similar treatment of a NT-deficient cell line resulted in increased efficacy as well. These results provide the basis for a future use of phosphorylated nucleoside analogues in cancer therapy.

## Methods

### Materials and reagents

Reagents and solvents were obtained from commercial sources and were used without further purification. All solvents used were HPLC quality (Carlo Erba reagents). Milli-Q water was obtained from a Millipore apparatus with a 0.22 μm filter. Phosphate buffer saline (PBS, Dulbecco’s phosphate buffer saline free of CaCl_2_ and MgCl_2_, 9.5 mM, Lonza), RPMI (Roswell Park Memorial Institute, basal medium) were used as drug release or cell culture media. Borate buffer (0.19 M, pH 7.32) was prepared using Na_2_B_4_O_7_.10H_2_O (0.05 M, 5.72 mg) and H_3_BO_3_ (0.2 M, 4.7 mg) in 5 mL D_2_O. Ammonium hydroxide 30 wt % water solution (Acros Organics) and acetic acid:triethylamine 2 M:2 M (TEAA, Aldrich) were employed. Gemcitabine (dFdC) hydrochloride (Sequoia Research Products Ltd, Toronto Research Chemicals) and dFdCMP and bis(triethylamine) dFdCTP (Toronto Research Chemicals) were used in part; bisammonium dFdCMP for NMR and UV-Vis titrations and release experiments was prepared as published recently^9^. The sample of tris(triethylammonium) dFdCTP used in NMR and release experiments was a gift of the National Institute of Cancer, NCI/NIH, USA^18^. Radiolabeled gemcitabine monophosphate ammonium salt [dFdCMP 5-^3^H(N)], was purchased from Moravek, Biochemicals Inc. packaged in sterile water at a concentration of 382.2 μg/mL, 2.0 mCi/mL (specific activity 2.3 Ci/mmol). Radiochemical purity was 98.7%. BODIPY® TR adenosine 5′-triphosphate (BODIPY® TR ATP) was purchased from Thermofischer. CD derivatives **1**, **2** and **3** and **1-FITC** were synthesized using a previously published procedure^12, 14^.

### Characterization methods

^1^H, ^19^F and ^31^P-NMR spectra and 2D NMR experiments were recorded on Bruker Avance 400 or 500 MHz instruments using D_2_O as solvent at 25 °C or deuterated borate buffer, pH 7.2 (pD 7.62).

HPLC measurements were performed on a Shimadzu HPLC system composed of a SPD-10UV-Vis detector, a LC-10AT VP pump with a DGU-14A degasser and a C18 Supelco HPLC column [(L × OD) = 25 cm × 4.6 mm, with 5 μm particle size]. Injection volumes were 20 μL. The detection wavelength was set at λ = 254 nm. The mobile phase was acetonitrile: water (75:25, *v/v*) at a flow rate of 0.5 mL/min.

### Theoretical methodology

The semi-empirical PM7 level of theory^26^ was employed as exists in the MOPAC program^27^. Solvent effects were implicitly treated by the conductor-like screening model (COSMO)^27^, using an effective radius of 1.3 Å and a dielectric constant of 78.4 for water. The geometries of all molecules were fully optimized leading to enthalpies of formation and finally to reaction enthalpies at 298 K with a cumulative uncertainty^28^ of 25 kJ mol^−1^. A large number of critical conformations for dFdCMP and **1** were constructed and optimized in order to identify the energetically lowest structures corresponding to their most likely conformations in water. All possible arrangements between host and guest molecules were considered, by placing the dFdCMP either: a) on the side of **1** away from cavity openings, b) outside either CD opening, or c) by insertion of dFdCMP inside the CD cavity *via* either opening.

### Release studies using a Franz cell setup

The setup comprised two cells (3.5 mL each, thermostated at 37 °C ± 0.5 °C) used in parallel for each case to measure the diffusion of dFdCMP (or dFdCTP) alone and in the presence of host **1**. The cells were separated by a benzoylated cellulose membrane (MWco = 1200, Sigma). The complexes dFdCMP/**1** and dFdCTP/**1** were prepared in advance in PBS (pH = 7.4) and were kept at 4 °C overnight, then allowed to reach the cell temperature before their introduction to the donor (D) compartment. The receptor (R) compartment was initially filled with PBS and at fixed time intervals (60, 120, 180, 240, 300 and 360 min) volumes were collected and immediately replaced with equal volumes of fresh PBS solution maintained at 37 °C ± 0.5 °C. The concentrations of the released guests in the collected R solutions were assessed by HPLC.

### Cell culture studies

MCF-7 (breast adenocarcinoma cell line) and CCRF-CEM (human leukaemia cell line) cells were obtained from the American Type Culture Collection (ATCC, Manassas, VA, USA). Human leukaemia cell line CCRF-CEM Ara-C/8 C, an Ara-C resistant cell line derived from the CCRF-CEM cell line, was kindly provided by Dr. Buddy Ullman (Oregon Health Sciences University). CCRF-CEM and CCRF-CEM Ara-C/8 C were cultured in RPMI 1640 medium. MCF7 cells were was cultured in DMEM medium, containing 10% fetal bovine serum (FBS), 100 U/mL penicillin G sodium and 100 mg/mL streptomycin sulfate (complete medium), in a humidified atmosphere of 95% air: 5% CO_2_ at 37 °C. Trypan Blue solution and trypsin were purchased from CellGro (Kansas City, MO). CellTiter-Blue® reagent (CTB) purchased from Promega Corporation (Wisconsin, USA) and methyl thiazolyl diphenyl-tetrazolium bromide (MTT, Sigma Aldrich, Saint-Quentin-Fallavier, France) test were used to assess cell viability (for details see SI). MCF7 cells were cultured in a mixture of DMEM/Ham’s F12 (1:1) media. All media were supplemented with 10% heat inactivated fetal bovine serum (FBS) (56 °C, 30 min), penicillin (100 U mL^−1^), streptomycin (100 mg mL^−1^) and L-glutamine (2 mM). Cells were maintained in a humid atmosphere at 37 °C with 5% CO_2_.

### Cell internalization assays

To determine the cell capture of the BODIPY^®^ TR ATP/**1-FITC** complexes, MCF7 cells were cultured on 12-well plates for 24 h to achieve 60−80% confluence. Free BODIPY^®^ TR ATP and BODIPY^®^ TR ATP/**1-FITC** at a 1:1 molar ratio were then added at the concentration of 1 μM to each well. After incubation, the cells were collected at different time intervals (*e*.*g*., for 1, 2.5, 5 and 24 h) for measurement of BODIPY^®^ TR fluorescence. The fluorescence from individual cells was examined using a flow cytometer C6 (Accuri Cytometers Ltd., UK). For the detection of BODIPY fluorescence, an argon laser was used (excitation at 543 nm) and the fluorescence emission was measured at 616 nm; 10000 cells were measured in each sample. The effect of cellular metabolism on cellular uptake of BODIPY^®^ TR ATP/**1-FITC** was assessed by incubating MCF7 cells at 4 °C prior to and during treatment with the nanocarriers. MCF7 cells were treated for 5 h with BODIPY^®^ TR ATP/1-FITC at either 37 °C or 4 °C, and the mean fluorescence intensities corresponding to cellular uptake were quantified using flow cytometry.

To study the cell localisation of BODIPY^®^ TR ATP/**1-FITC** complexes, MCF7 cells were cultured on a coverslip in a culture dish for 24 h to achieve approximately 40% confluence. Cells were then incubated with BODIPY^®^ TR ATP and BODIPY^®^ TR ATP/**1-FITC** at the concentration of 1 μM at 37 °C for 24 h. After washing five times with Dulbecco’s PBS, the cells were observed in a confocal microscope (Zeiss) with a × 60 oil-immersion objective. The following wavelengths were used: excitation at 543 nm and detection through a 616 nm filter for BODIPY^®^ TR and excitation at 488 nm and detection through a 515 nm filter for FITC.

### Cell internalization experiments using radioactive ^3^H- dFdC/**1** complexes

MCF7 cells were cultured in a humid atmosphere at 37 °C with 5% CO_2_ on 6 well plates for 24 h to achieve confluence. Experiments were carried out with radiolabeled dFdCMP, using a stock solution prepared by mixing non-radioactive dFdCMP and 1% of dFdCMP [^3^H]. Samples of 100 μL of dFdCMP or dFdCMP/**1** at 1 µM were added to the wells and incubated up to 24 h. After incubation, the supernatants and the cells were collected at different time intervals (i.e., 0.5, 1, 2.5, 5 and 24 h). Radioactivity was counted using a Beckman Coulter apparatus (LS 6500 multipurpose scintillation counter) in the supernatants to determine the amount of non-internalized dFdCMP and in the lysed cells. All experiments were performed in triplicate. Data were reported as mean ± standard deviation and analyzed by single-factor ANOVA, setting the level of significance at p < 0.05.

### Statistical analysis

For comparison of several groups, one-way ANOVA with Bonferroni correction was performed using GraphPad Prism version 5.0 software (GraphPad Software, Inc, CA, USA). All numerical data were expressed as mean ± SD, n = 3 or 4, from 3 different experiments. Any p values ≤ 0.05 was considered statistically significant.

## Electronic supplementary material

Supplementary information
