# Supplementary material for: Positively charged cyclodextrins as effective molecular transporters of active phosphorylated forms of gemcitabine into cancer cells
Source: Sci Rep. 2017 Aug 21;7:8353. doi: 10.1038/s41598-017-08727-y (PMC5566897; doi:10.1038/s41598-017-08727-y)
Supplement: Supplementary file 1 — Supplementary information [file 41598_2017_8727_MOESM1_ESM.doc]

**Supporting Information**

**Original article**

**Positively charged cyclodextrins as effective molecular transporters of active phosphorylated forms of gemcitabine into cancer cells**

Violeta Rodriguez-Ruiz^a,&^, Andrey Maksimenko^a,b,&^, Giuseppina Salzano*^c^*, Maria Lampropoulou^d^, Yannis G. Lazarou^d^, Valentina Agostoni^a^, Patrick Couvreur^a^, Ruxandra Gref^a,c^* and Konstantina Yannakopoulou^d*^

*^a^Institut Galien (UMR CNRS 8612), Université Paris-Sud, Châtenay-Malabry, France*

*^b^UMR CNRS 8200, Gustave Roussy, DNA repair group, F-94051, Villejuif, France*

*^c^Institut des Sciences Moléculaires d'Orsay (UMR CNRS 8214), Université Paris-Sud, Université Paris-Saclay, Orsay, France*

*^d^National Center for Scientific Research “Demokritos”, Institute of Nanoscience & Nanotechnology, Ag. Paraskevi 15310, Athens, Greece*

Current addresses:

Violeta Rodriguez-Ruiz: *Université de Cergy Pontoise, ERRMECe, Biomaterials for Health group, I MAT, F-95302, Cergy Pontoise, France*

*Corresponding authors: E-mail addresses: [ruxandra.gref@u-psud.fr](mailto:ruxandra.gref@u-psud.fr) and [k.yannakopoulou@inn.demokritos.gr](mailto:dyanna@chem.demokritos.gr)

^&^V.R.-R. and A.M. contributed equally to this work.


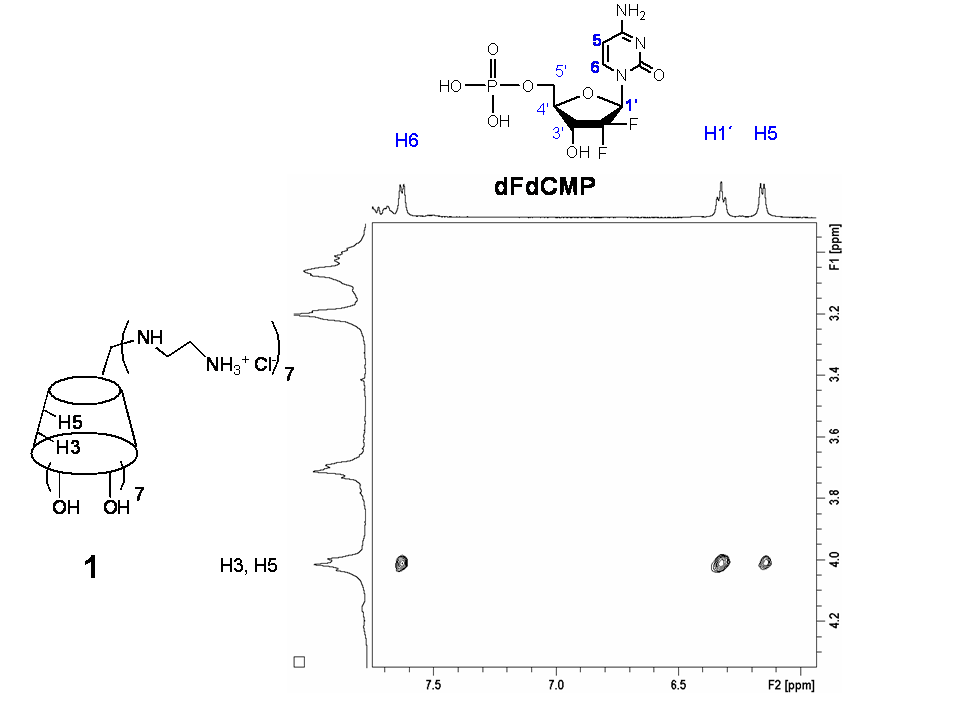


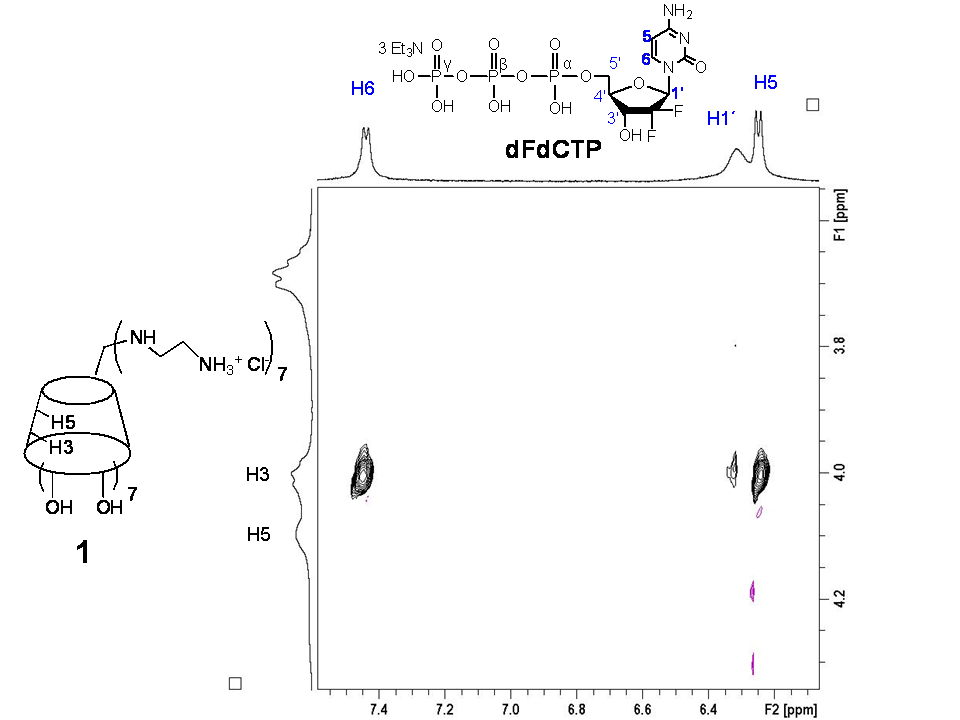


**Figure S1.** 2D ROESY spectrum of (a) dFdCMP/**1** (6.4 mM/6.4 mM, borate buffer in D_2_O, 298K, pH =7.3) and (b) of dFdCTP/**1** (4 mM/ 4 mM, D_2_O), 500 MHz, 298K.


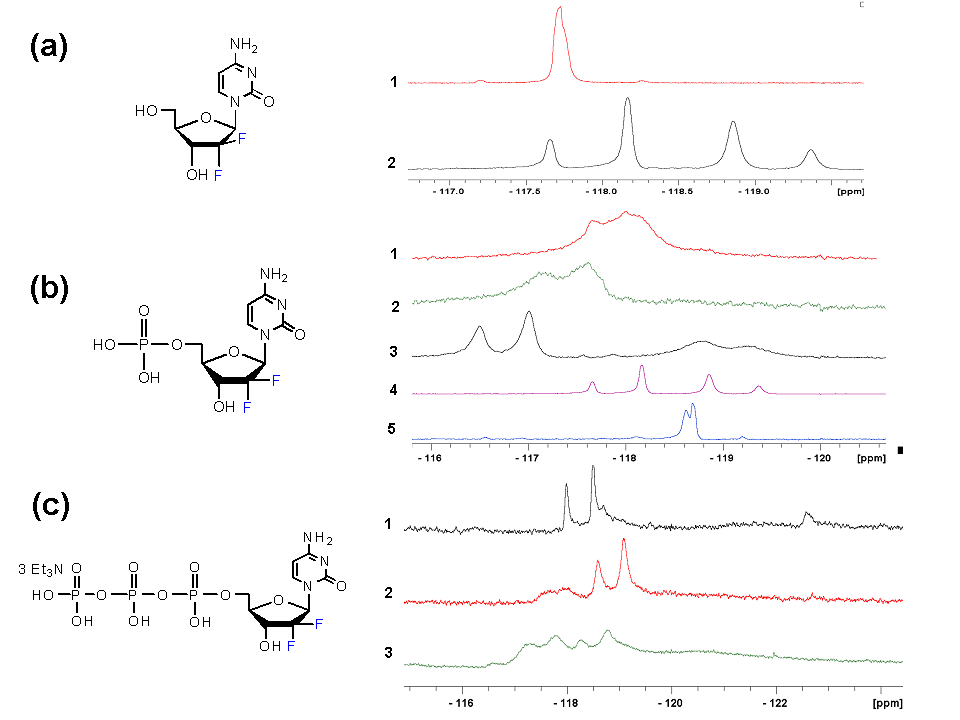


**Figure S2.** ^19^F NMR spectra in borate buffer in D_2_O of a1) dFdC alone a2) dFdC/βCD (7.4 mM/7.4 mM); b1) dFdCMP**/3** (6.4 mM/6.4 mM) b2) dFdCMP**/2** (6.4 mM /6.4 mM) b3) dFdCMP**/1** (6.4 mM /6.4 mM, b4) dFdCMP**/**βCD (6.4 mM /6.4 mM), b5) dFdCMP alone in borate buffer in D_2_O; c1) dFdCMP**/3**, c2) dFdCMP**/2**, c3) dFdCMP**/1**.

**Theoretical Calculations**

The theoretical calculations were carried out by using the MOPAC suite of programs (MOPAC2012, Version 13.085L)^1^ at the PM7 level of theory^2^ including the treatment of solvent effects by the COSMO methodology^3^.

***Guest dFdCMP***

The acid-base equilibria of the guest molecule, dFdCMP, are governed by the two acidic hydrogen atoms of the phosphate group and the most basic heteroatom (nitrogen or oxygen) of cytosine group. The corresponding ionization constants of the phosphate group are expected to be similar to those of dCMP, whose pKa were reported to be 0.7 ± 0.3 and 6.55 ± 0.02 ^4^. The pKa of dFdC was reported to be 3.6 ^5^, whereas the presence of the phosphate group is expected to increase the pKa of cytosine in dFdCMP to 3.9 (ΔpKa = 0.3), by comparing the corresponding pKa values for dC and dCMP ^6^. Therefore, in aqueous solution dFdCMP may speciate as four different forms: cation dFdCMPH^+^ (protonated cytosine), neutral zwitterion dFdCMP^±0^ (protonated cytosine with singly deprotonated phosphate), monoanion dFdCMP^-1^ (singly deprotonated phosphate), and dianion dFdCMP^-2^ (doubly deprotonated phosphate). Considering the values 0.7, 6.5 and 3.6 for the pKa of phosphate group and cytosine, respectively, the relative concentrations for the four different forms were calculated as a function of the pH. The results suggest that at a pH of 7.3, dFdCMP is exclusively present as dFdCMP^-1^ and dFdCMP^-2^, with populations of 15% and 85%, respectively.

For both anion forms of dFdCMP the conformational search considered the *syn*- and *anti*- conformations for cytosine and the C3'-endo (N) or the C2'-endo (S) puckering of the ribose ring. The conformational freedom around the C4'-C5' bond was also considered, leading to three staggered conformations, namely gauche-gauche (gg), gauche-trans (gt) and trans-gauche (tg) with a dihedral C3'-C4'-C5'-O5_P_ angle of 60, 180 and -60 degrees, respectively. The lowest energy conformers are shown in Figure 1 whereas typical higher-energy conformers are shown in Figure S3.

| 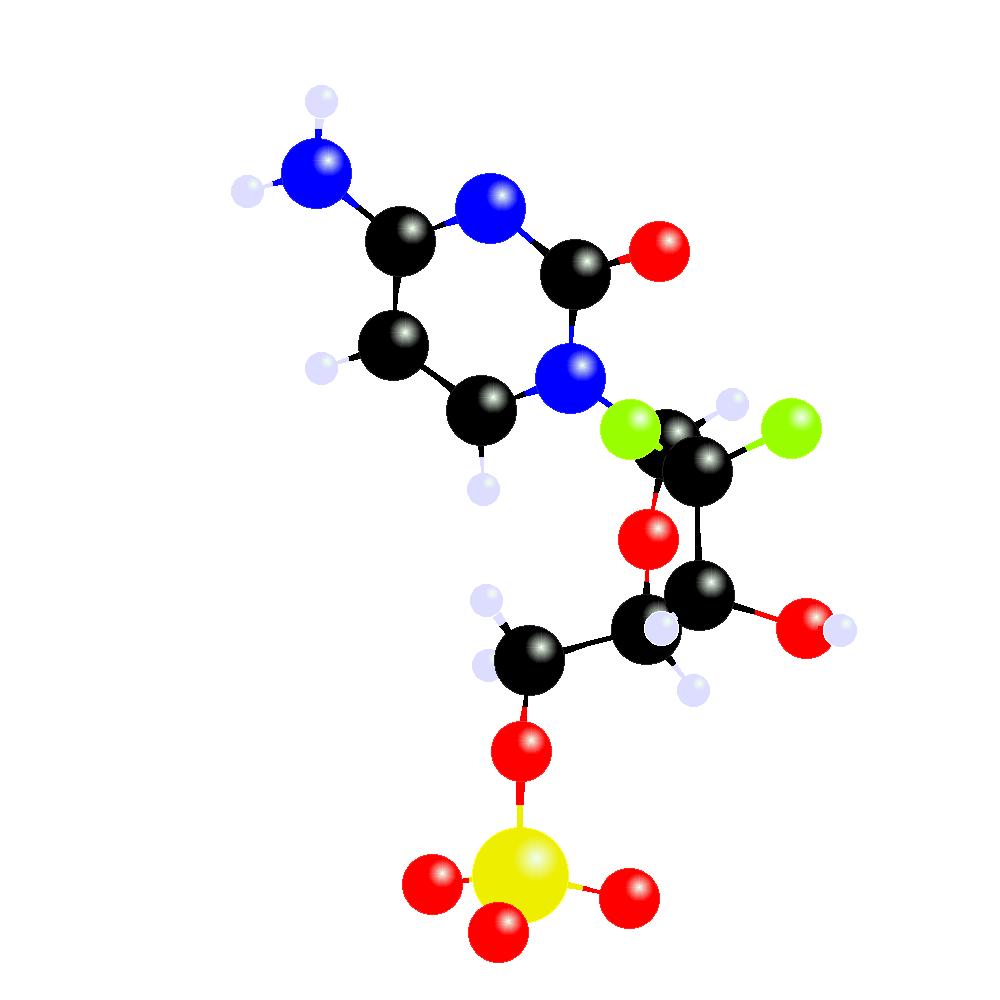 | 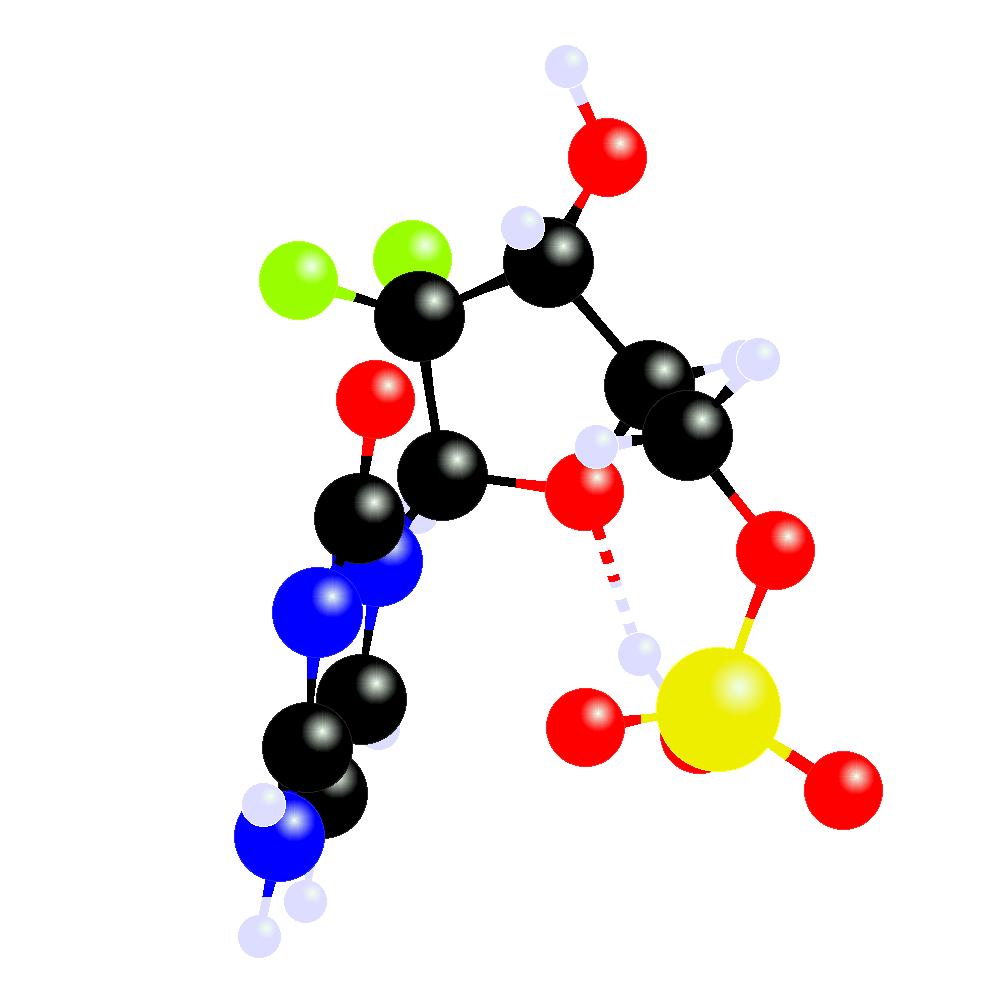 |
| --- | --- |
| **A** (0.9) | **C** (0.3) |
| 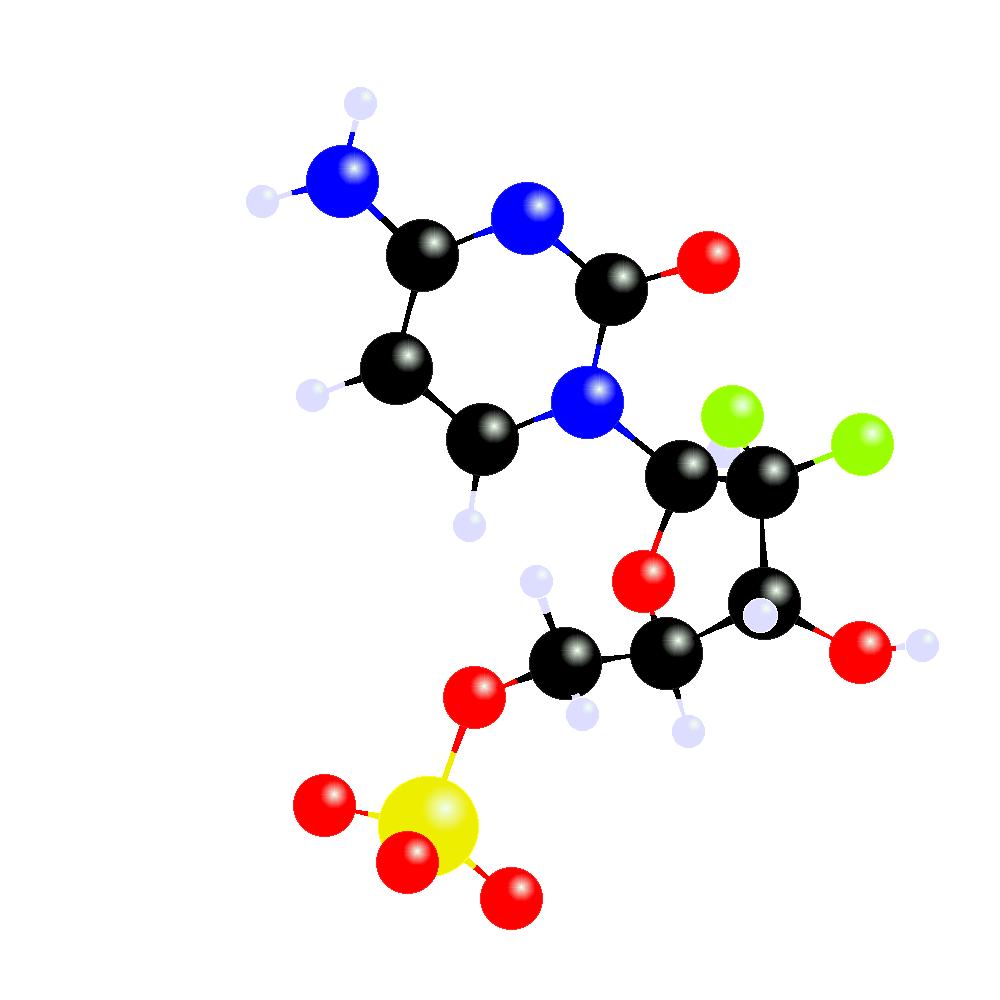 | 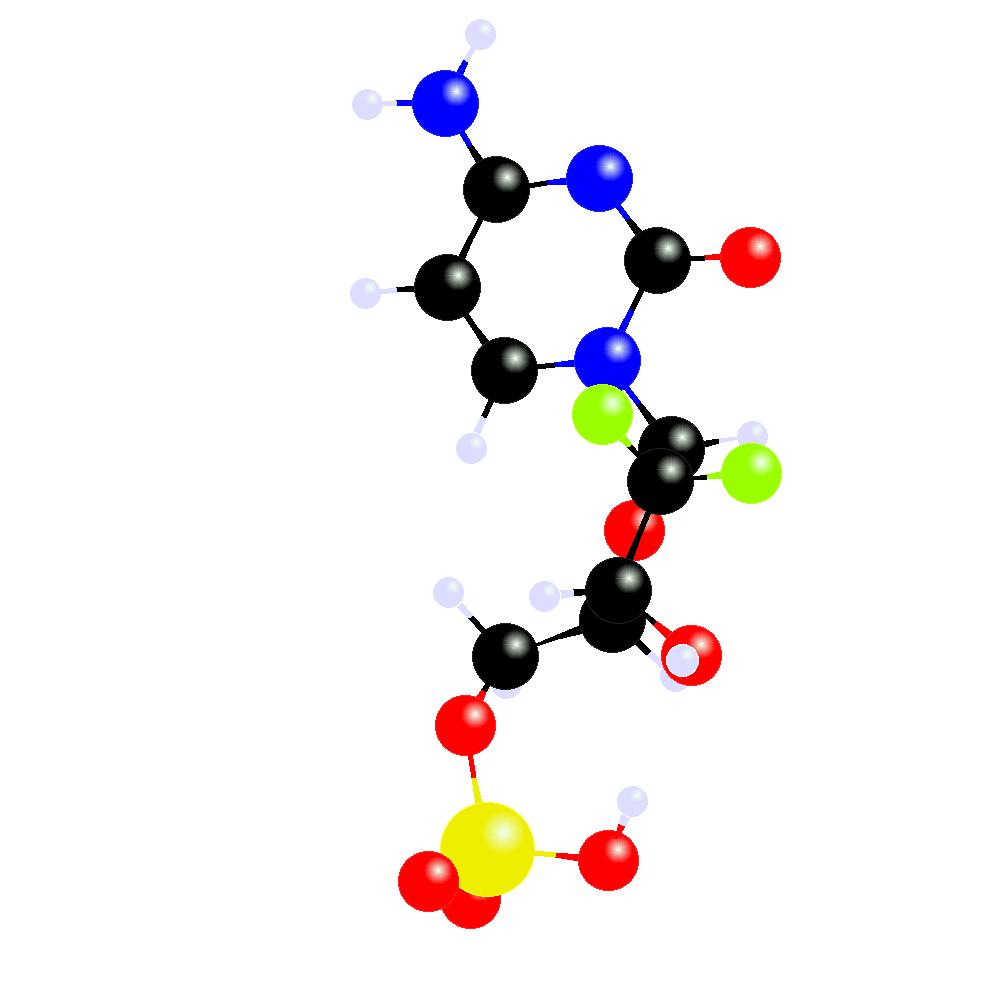 |
| **B** (7.5) | **D** (5.2) |

**Figure S3.** Typical low energy conformers of dFdCMP^-2^ (**A, B**) and dFdCMP^-1^ (**C, D**) at the PM7-COSMO level of theory. Values in parentheses denote the enthalpy differences (kJ mol^-1^) from the most stable conformers, shown in Figure 2. Color coding: C (black); H (off-white); N (blue); O (red); F (green); P (yellow).

The energy difference between C2' and C3' endo conformations of ribose was found to be very sensitive on the relative conformations of the other flexible groups in the molecule, in particular on the conformation of cytosine. For *syn*- conformations of cytosine the repulsive steric interactions of its O_2_ atom with the fluorine atoms imparts almost exclusively a C3' endo conformation of the sugar moiety, The phosphate group orientation in dFdCMP^-1^ may also affect the puckering of the ribose moiety, via the POH...O3' hydrogen bond whose formation is favoured in a C3' endo conformation. The most stable form of dFdCMP^-1^ possesses a C2' endo conformation (Figure 2), whereas a C3' endo conformation is dominant for the remaining less stable forms up to 5 kJ mol^-1^. For dFdCMP^-2^, C3' endo conformations are also favoured for the more stable conformers up to 3.2 kJ mol^-1^. For the most stable conformer of dFdCMP^-1^, an *anti*- cytosine arrangement is noted, although syn-cytosine arrangements are also possible for the less stable forms within a small energy range of 3.5 kJ mol^-1^. Similarly, *anti*- cytosine arrangements are preferred for the lowest energy conformers of dFdCMP^-2^, with *syn*- arrangements equally possible within 1.5 kJ mol^-1^. For dFdCMP^-1^, the C3' hydroxyl group is oriented towards a F atom with H...F distances in the range 2.61 - 2.85 Å. For dFdCMP^-2^ and in particular for tg conformations, the OH group is preferably oriented towards the phosphate group to form a OH...O hydrogen bond with H...O distances around 1.7 Å.

***Host 1***

The host molecule **1**, contains seven primary (α) and seven secondary (ω) amino groups, whose pK_a_ were experimentally determined by ^13^C NMR tritrarion experiments^7^ to 9.5 and 6.4, respectively, or 4.5 and 7.6 in terms of the corresponding pK_b_. The counterintuitive higher basicity of the primary amino groups can be attributed to the longer mutual separations attainable (due to the flexibility of the alkyl chains) leading to weaker electrostatic repulsions of the corresponding -NH_3_^+^ groups.

The relative concentrations of the two forms of **1** in aqueous solution were estimated by considering the equilibria:

**1** + 7 H_2_O <======> **1**H_7_^7+^ + 7 OH^-^ (K_bprim_) (1)

**1**H_7_^7+^ + 7 H_2_O <======> **1**H_14_^14+^ + 7 OH^-^ (K_bsec_) (2)

Due to the large cyclodextrin size and the corresponding inter-units separation, the successive 7 protonation steps to the primary nitrogen atoms are assumed to possess similar equilibrium constants K_bprim1_, K_bprim2_, ..., K_bprim7_, each roughly equal to10^-4.5^:

K_bprim1_ = [**1**H^+^] [OH^-^] / [**1**] ≈ 10^-4.5^

K_bprim2_ = [**1**H_2_^2+^] [OH^-^] / [**1** H^+^] ≈ 10^-4.5^

K_bprim3_ = [**1**H_3_^3+^] [OH^-^] / [**1** H_2_^2+^] ≈ 10^-4.5^

.....

K_bprim7_ = [**1**H_7_^7+^] [OH^-^] / [**1** H_6_^6+^] ≈ 10^-4.5^

Insertion of the above into equation (1) with successive substitutions leads to:

K_bprim1_ K_bprim2_ ... K_bprim7_ = [**1**H_7_^7+^] [OH^-^]^7^ / [**1**] ≈ (10^-4.5^)^7^ (3)

In a similar manner, the following equation is obtained for K_bsec_:

K_bsec1_ K_bsec2_ ... K_bsec7_ = [**1**H_14_^14+^] [OH^-^]^7^ / [**1** H_7_^7+^] ≈ (10^-7.6^)^7^ (4)

Finally, let C be the initial concentration of **1**, of which x1 concentration units yield **1**H_7_^7+^, whereas x2 units of **1**H_7_^7+^ are lost to **1**H_14_^14+^. Therefore, at equilibrium, equations (3) and (4) become

K_bprim_^7^ = ((x1 - x2) [OH^-^]^7^ ) / (C - x1) (5)

K_bsec_^7^ = (x2 [OH^-^]^7^ ) / (x1 - x2) (6)

Since K_w_ = [H^+^][OH^-^] for aqueous solutions, equations (5) and (6) become:

K_bprim_^7^ = (x1 - x2) (K_w_^7^ / [H^+^]^7^) / (C - x1) (7)

K_bsec_^7^ = (x2) (K_w_^7^ / [H^+^]^7^) / (x1 - x2) (8)

For a constant [H^+^], equations (7) and (8) constitute a system of two equations with two unknowns (x1 and x2, in terms of the starting concentration of **1,** C). Setting [H^+^] = 1 x 10^-7.3^ and C = 1, the system yields x1 ≈ 1.0 and x2 = 5 x 10^-7^. The result implies that in a neutral solution, the host molecule exists almost entirely as a +7 cation (7 protons on the primary nitrogen atoms).

An extensive conformational search was performed for **1**, protonated on either the primary or the secondary amino groups, in order to benchmark the performance of the PM7(COSMO) level of theory in predicting the most basic type of nitrogen atoms in aqueous solution. The search included the rotation of all seven -C6H_2_NHCH_2_CH_2_NH_3_^+^ groups around the C5-C6 axis leading to the gauche-gauche (all-gg) and gauche-trans (all-gt) conformers. In addition, all possible staggered conformers resulting from the rotation of the C-C and C-N bonds within the -NHCH_2_CH_2_NH_3_^+^ group were considered. The most stable species possesses a symmetric all-gg conformation (Figure S4A) and lies only 0.4 kJ mol^-1^ lower in energy than the most stable mixed gt/tg conformer which contains a –C6H_2_NHCH_2_CH_2_NH_3_^+^ chain located nearly inside the cyclodextrin (Figure S4B).

| 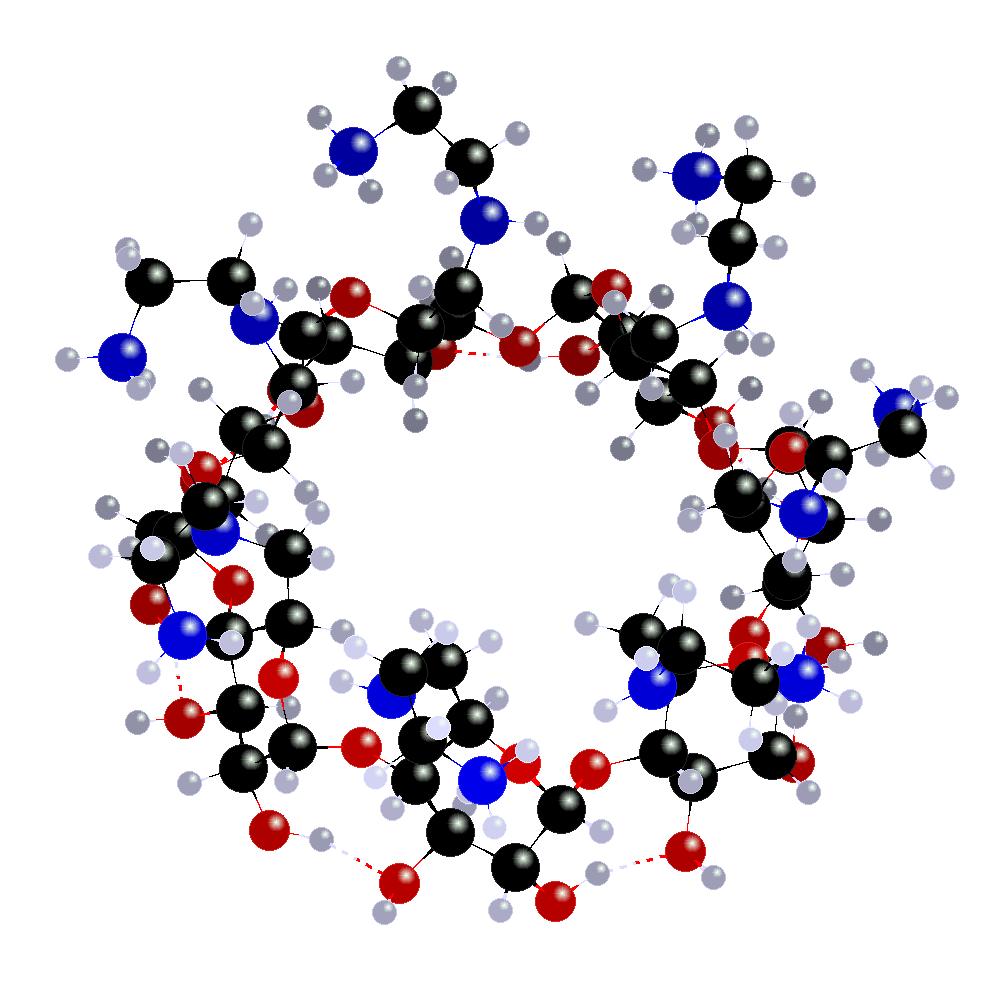  **A** | 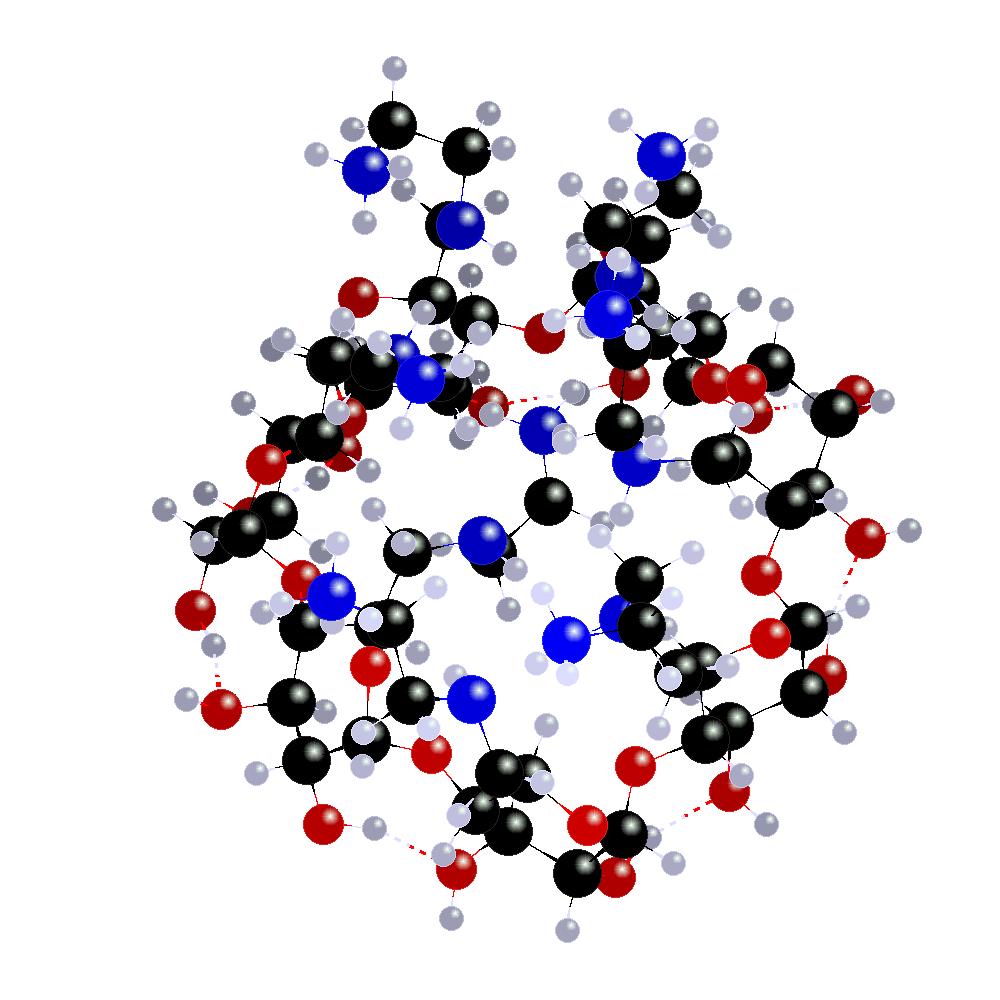  **B** |
| --- | --- |

**Figure S4.** Lowest-energy conformers of **1**H_7_^+7^: (**A**) All-gg conformer and (**B**) mixed gt/tg conformer. Color coding: C (black); H (off-white); N (blue); O (red).

*dFdCMP****/1 Complexes***

A subset of the lowest energy conformers for both dFdCMP^-1^ and dFdCMP^-2^ along with the lowest energy all-gg, mixed gg/gt and mixed gt/tg conformers of 1H_7_^7+^ was employed to construct the initial geometries of complexes which were fully optimized at the PM7-COSMO level of theory. A plethora of mutual arrangements was considered, by placing the dFdCMP species on the exterior surface of the cyclodextrin, lying above its primary or secondary sides as well as inside its interior in a variety of relative orientations.

The structural parameters and the stability of these complexes are primarily governed by electrostatic interactions between the negatively charged phosphate group of dFdCMP and the positively charged -NH_3_^+^ groups of 1H_7_^7+^, in addition to various intermolecular non-bonding interactions. Two main types of strong complexes may be sorted out, which involve:

1) Association of dFdCMP to the primary side of mixed gt/tg conformers of 1H_7_^7+^, driven by attractive electrostatic interactions between the -OPO­_3_H^-^ / -OPO_3_^-2^ and -NH_3_^+^ groups and the formation of intermolecular hydrogen bonds. The shortest O_P_-N distances observed are in the order of 2.6 Å. The corresponding geometries for the lowest-energy association complexes of dFdCMP^-1^ and dFdCMP^-2^ are shown in Figure S5 A,C.

2) Inclusion of dFdCMP in the cyclodextrin cavity of all-gg or mixed gg/gt conformers of **1**H_7_^7+^, appropriately oriented as a result of the attractive interactions between the oppositely charged groups. The stability of inclusion complexes was calculated to be greater for mixed gg/gt than all-gg conformers of 1H_7_^7+^, due to the favourable proximity of phosphate group to -NH_3_^+^ groups on side chains possessing the gt conformation. The corresponding geometries for the lowest-energy inclusion complexes of dFdCMP^-1^ and dFdCMP^-2^ are shown in Figure S5 B,D.

However, the calculated enthalpy differences among these two types of complexes are generally small, amounting to less than 10 kJ mol^-1^ for the most stable complexes for each dFdCMP anionic form and therefore, they may coexist in aqueous solution.

| 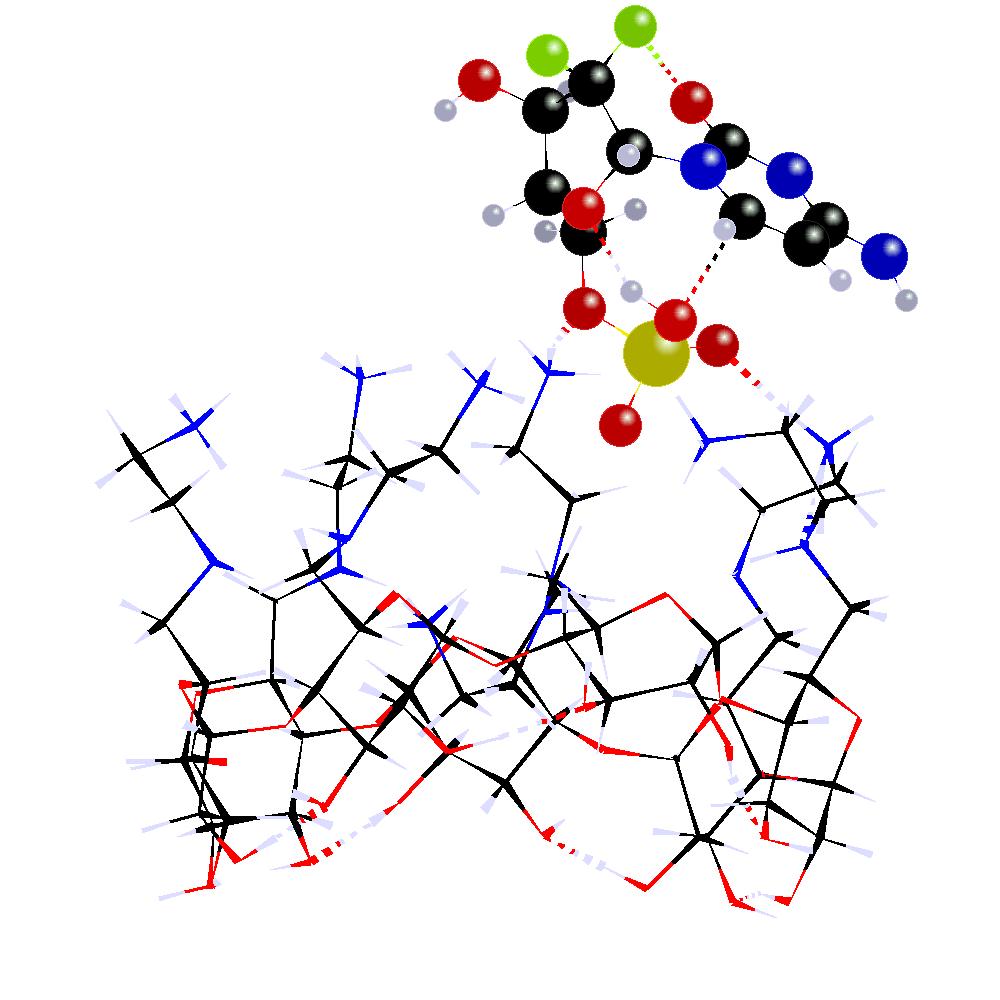 | 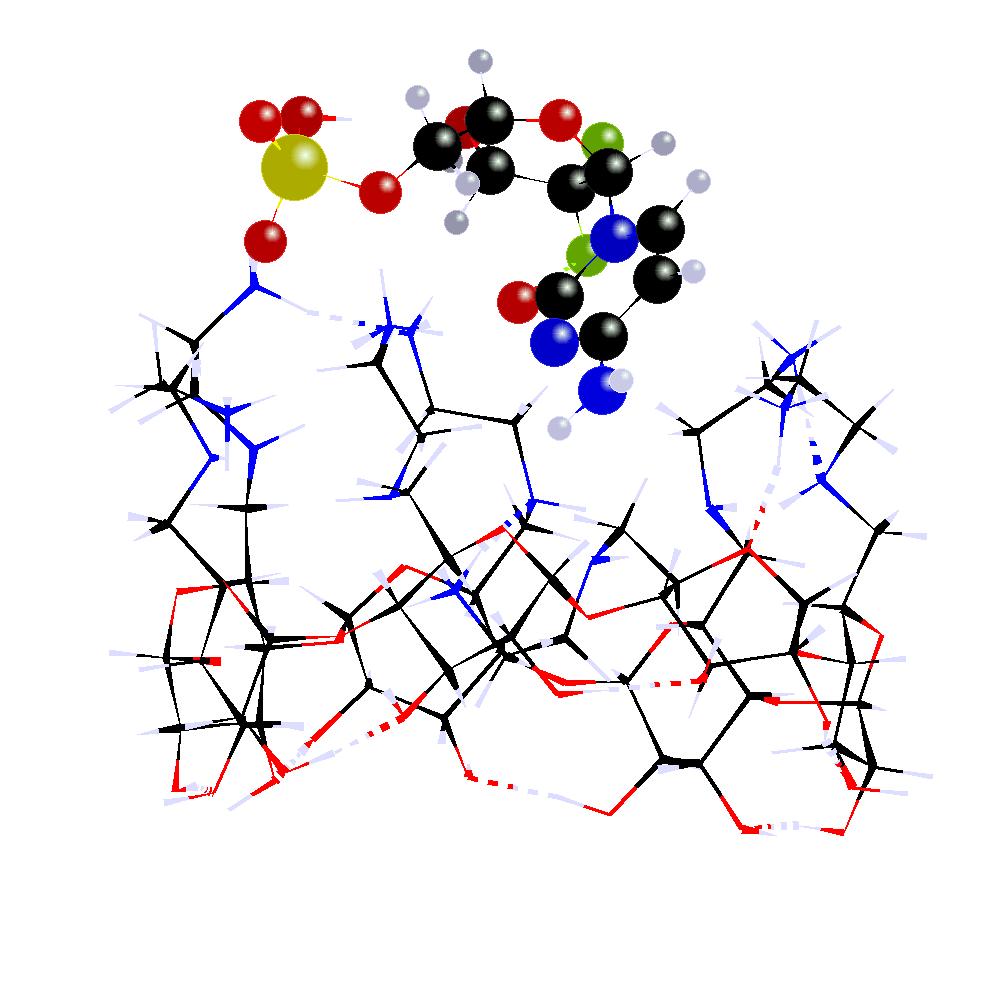 |
| --- | --- |
| **A** (9.0) | **C** (0.0) |
| 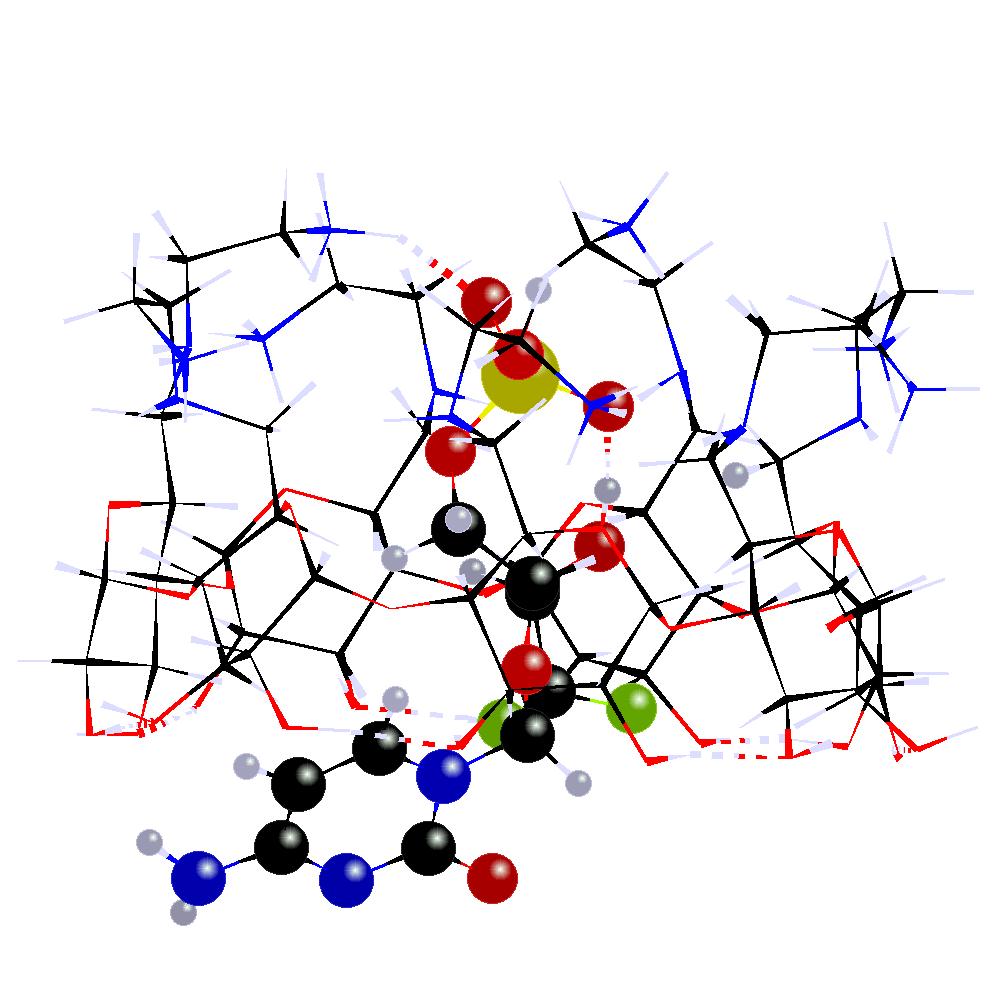 | 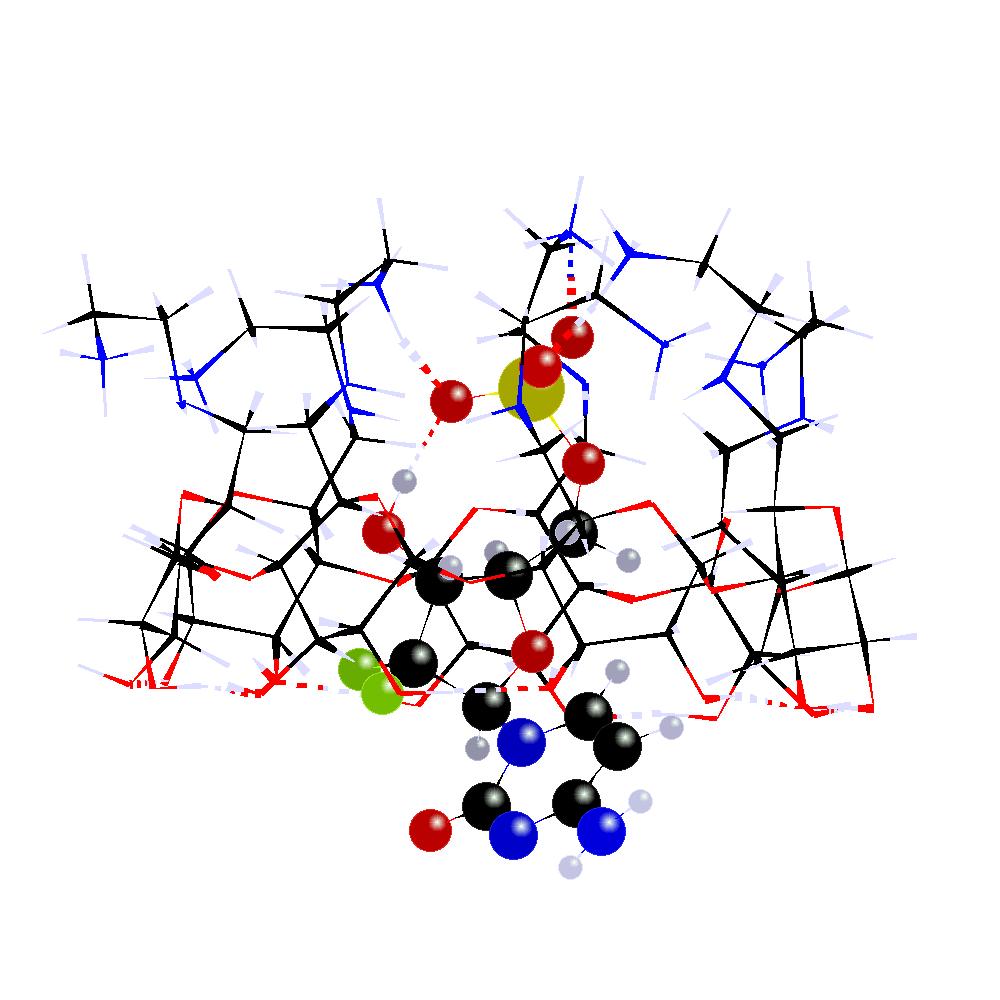 |
| **B** (0.0) | **D** (6.7) |

**Figure S5.** Additional lowest-energy association (top) and inclusion (bottom) complexes of **1**H_7_^7+^ with dFdCMP^-1^ (**A, B**) and dFdCMP^-2^ (**C, D**). Values in parentheses denote the enthalpy difference (in kJ mol^-1^) from the most stable complex for each anionic form. Color coding: C (black); H (off-white); N (blue); O (red); F (green); P (yellow).

**Table S1.** Distances (in Å) between H1', H5 and H6 of dFdCMP with cavity hydrogen atoms CDH3 and CDH5 of **1**H_7_^7+^ for the most stable 1:1 complexes of dFdCMP^-2^ and dFdCMP^-1^ as well as of 1:2 complexes of dFdCMP^-2^ with mixed gg/gt and gt/tg conformers of **1**H_7_^7+^. Distances less than 3.5 Å are shown in boldface.

|  |  | **H1'-CDH3** | **H1'-CDH5** | **H5-CDH3** | **H5-CDH5** | **H6-CDH3** | **H6-CDH5** |
| --- | --- | --- | --- | --- | --- | --- | --- |
|  |  |  |  |  |  |  |  |
| 1:1 dFdCMP^-1^ | **inclusion** | **3.2** | 4.0 | **2.0** | 3.9 | **2.3** | **2.7** |
|  | **association** | 11.2 | 9.8 | 10.1 | 8.6 | **2.8** | 6.0 |
|  |  |  |  |  |  |  |  |
| 1:1 dFdCMP^-2^ | **inclusion** | **3.2** | 4.7 | **2.1** | **3.4** | **3.2** | **2.5** |
|  | **association** | 12.1 | 8.7 | 8.1 | 7.0 | 9.9 | 8.3 |
|  |  |  |  |  |  |  |  |
| 1:2 dFdCMP^-2^ | **inclusion/**  **association** | **3.1** | 3.6 | 5.5 | **3.3** | 3.9 | **2.6** |


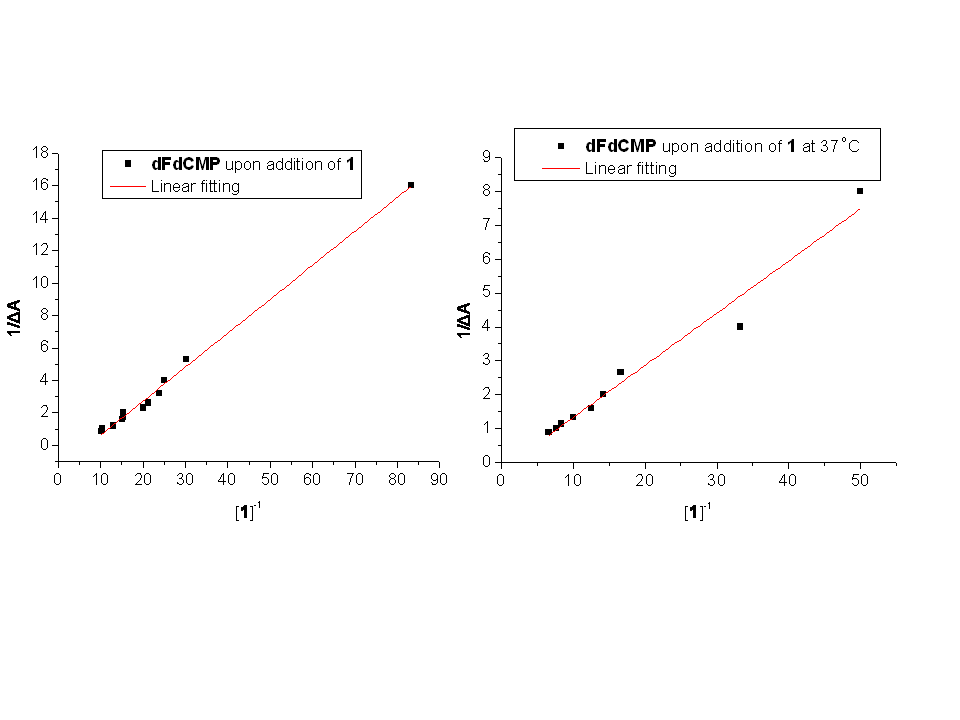


(a) (b)

**Figure S6:** Linear fitting of differences in absorbance of a) dFdCMP (0.0625 mM) upon addition of **1** (0.0625 mM to 1.1875 mM): K_assoc_ = 7.0 x 10^3^ M^–1^ ± 10%, (R^2^ =0.995), PBS, 22 ^o^C; b) of dFdCMP (0.0625 mM) upon addition of **1** (0.125 mM to 1.125 mM), K_assoc_ = 1.4 x 10^3^ M^–1^ ± 20%, (R^2^ =0.966), PBS, 37 ^o^C.


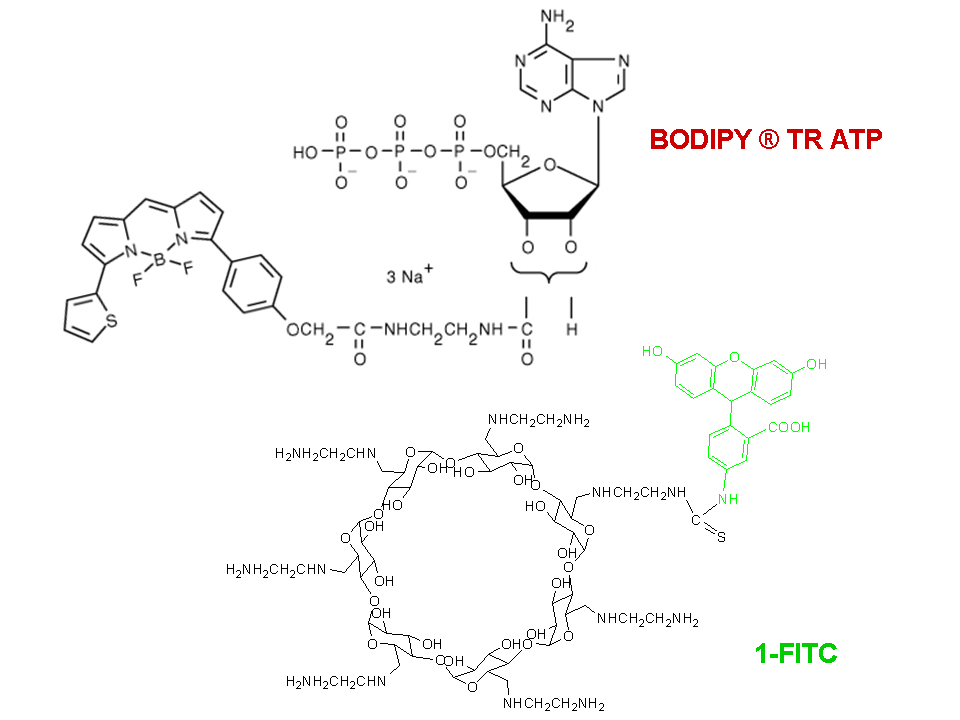


**Figure S7.** The structures of guest BODIPY TR ATP (upper, red fluorescence) and host **1**-FITC (lower, green fluorescence) used for cell internalization and confocal microscopy studies.

*Cell viability assays*

MTT assay: [3-(4,5-Dimethylthiazol-2-yl)-2,5-diphenyl tetrazolium bromide] was used to test cytotoxicity of active phosphorylated forms of dFdC encapsulated in CDs. Briefly, cells (*e.g.* MCF7, CCRF-CEM and CCRF-CEM Ara-C/8C) (5 × 10^3^/well) were seeded in 96-well plates. After overnight incubation, the cells were then exposed to a series of concentrations of dFdCMP/host **1**, dFdCTP/host **1**, host **1**, or free dFdCMP, dFdCTP and dFdC for 72 h. After drug exposure, the medium was removed and 100 μL of MTT solution (0.5 mg·mL^−1^ in DMEM containing 10% FBS) was added to each well. The plates were incubated for 2 h at 37 °C and 100 μL of 20% sodium dodecyl sulfate (SDS) solution was then added to each well for 24 h at 37 °C. Absorbance was measured at 570 nm and the percentage of surviving cells was calculated as the absorbance ratio of treated to untreated cells. The inhibitory concentration 50% (IC_50_) of the treatments was determined from the dose-response curve by noting the concentration at which the curve passes through the 50% inhibition level. All experiments were performed in quadruplicate to determine means and standard deviations (SDs).

CellTiter-Blue® assay: MCF7 cells were seeded in 96-well flat-bottomed cell culture treated plates at 1x10^4^ cells per solutions of dFdCMP or dFdCTP alone or in 1:1 complex with host **1** and also solutions of dFdC alone at concentrations ranging from 35 μM to 0.03 μM at 37 °C for 5 h in serum-free media followed by removal of the treatment and then incubation with fresh serum containing medium until the 72 h time-point was reached. Solutions of **1** alone at the same concentrations were used as the control experiment. After 72 h, the wells were washed twice with serum complete medium and cell viability was measured by the CellTiter-Blue® assay according to the manufacturer’s protocol. PBS treated cells were used as control and cell viability was plotted against concentrations of drugs. The 50% inhibitory concentrations (IC_50_) were determined from the dose-response curve. All treatments were carried out in triplicate for at least two independent experiments to determine means and standard deviations (SDs).


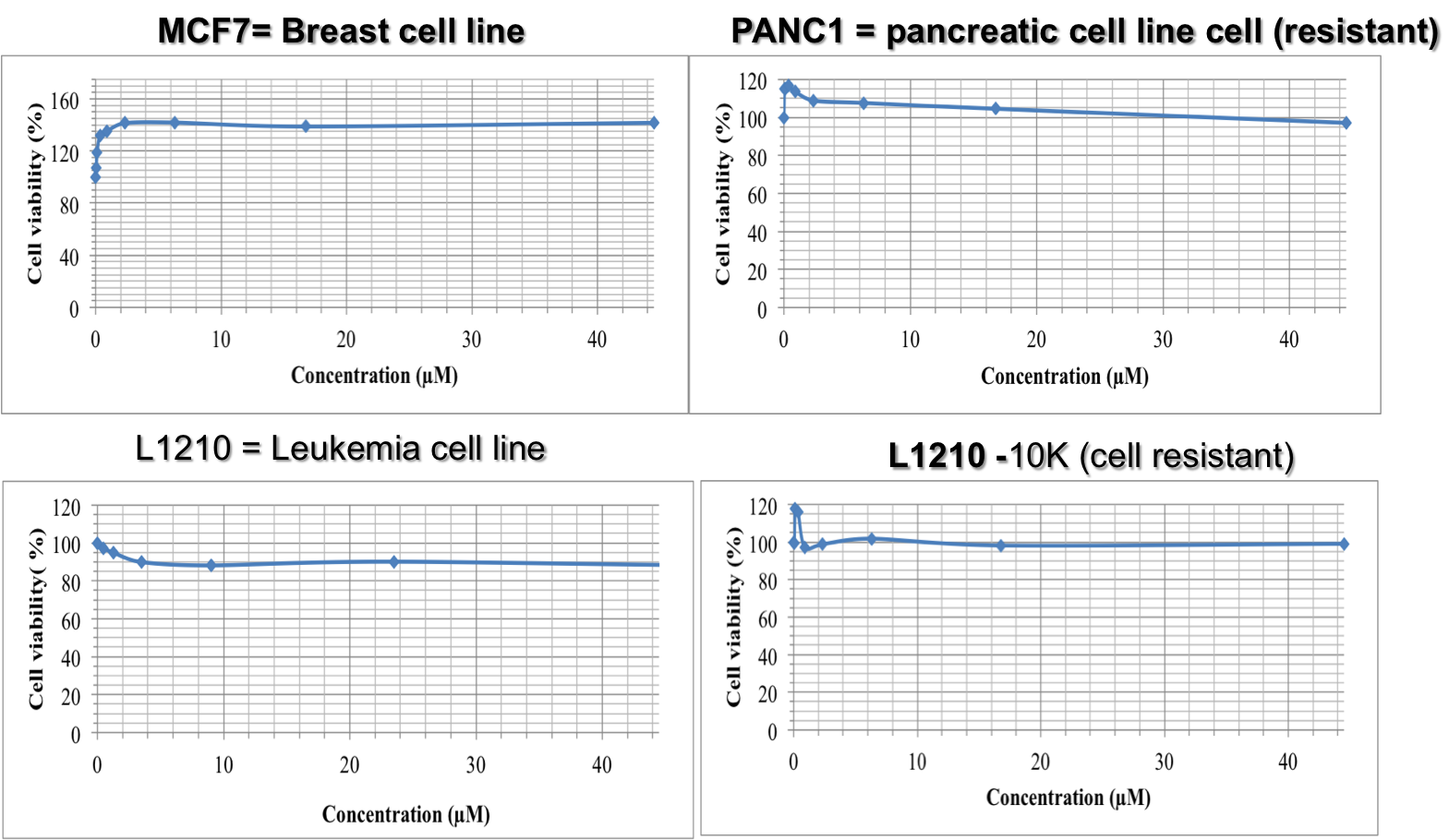

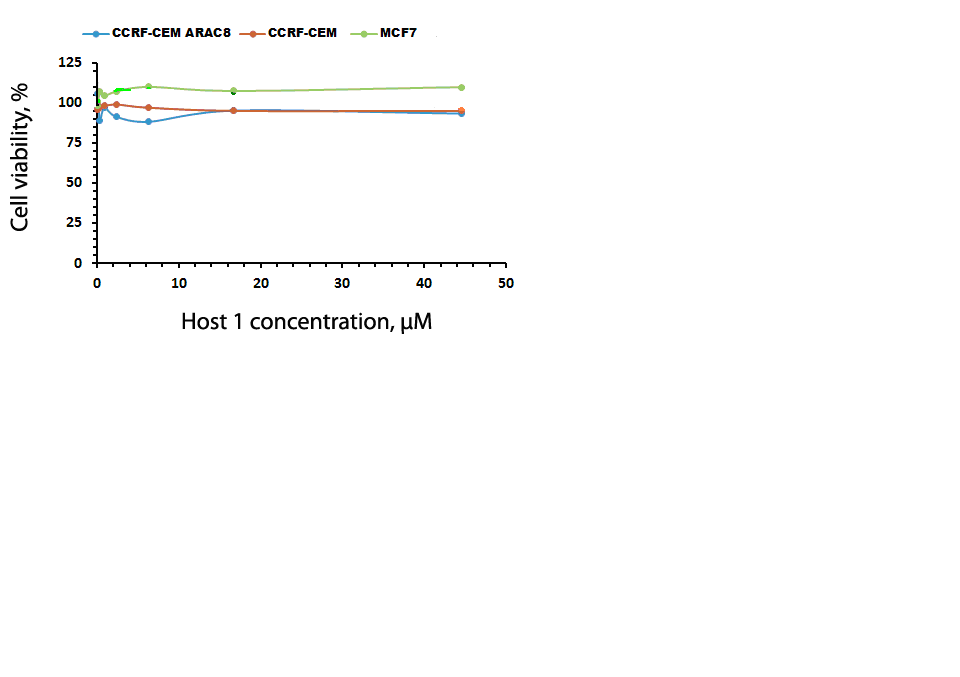


**A B**

**Figure S8**. Viability assays on MCF7, CCRF-CEM and CCRF-CEM Ara-C/8C cell lines with various concentrations of host **1**. **A**: CellTiter-Blue® assay, MCF7 cells; **B**: MTT assays.

Cell viability is preserved at all concentrations.

**References**
